# Optimization of Agricultural and Urban BMPs to Meet Phosphorus and Sediment Loading Targets in the Upper Soldier Creek, Kansas, USA

**DOI:** 10.3390/w17152265

**Published:** 2025-07-30

**Authors:** Naomi E. Detenbeck, Christopher P. Weaver, Alyssa M. Le, Philip E. Morefield, Samuel Ennett, Marilyn R. ten Brink

**Affiliations:** 1 Atlantic Coastal and Environmental Sciences Division, United States Environmental Protection Agency (US EPA), Narragansett, RI 02882, USA; 2 Integrated Climate Sciences Division, United States Environmental Protection Agency, Research Triangle Park, Durham, NC 27709, USA; 3 ICF International, Cambridge, MA 02451, USA; 4 Retired from Atlantic Coastal and Environmental Sciences Division, United States Environmental Protection Agency, Narragansett, RI 02882, USA

**Keywords:** optimization, watershed management, stormwater, agricultural runoff, best management practices

## Abstract

This study was developed to identify the optimal (most cost-effective) strategies to reduce sediment and phosphorus loadings in the Upper Soldier Creek, Kansas, USA, watershed using the Watershed Management Optimization Support Tool (WMOST) suite of programs. Under average precipitation, loading targets for upland total phosphorus (TP) could be met with use of grassed swales for treating urban area runoff and of contouring for agricultural runoff. For a wet year, the same target could be met, but with use of a sand filter with underdrain for the urban runoff. Both annual and daily TP loading targets from Total Maximum Daily Loads (TMDLs) were exceeded in simulations of best management practice (BMP) solutions for 14 alternative future climate scenarios. We expanded the set of BMPs to include stream bank stabilization (physical plus riparian restoration) and two-stage channel designs, but upland loading targets could not be met for either TP or total suspended solids (TSS) under any precipitation conditions. An optimization scenario that simulated the routing of flows in excess of those treated by the upland BMPs to an off-channel treatment wetland allowed TMDLs to be met for an average precipitation year. WMOST can optimize cost-effectiveness of BMPs across multiple scales and climate scenarios.

## 1. Introduction

States, tribes, and local watershed planning organizations are faced with the challenge of meeting nutrient and sediment loading targets both for present conditions and uncertain future climate scenarios. In the United States, watershed plans are developed to support implementation of loading targets, or total maximum daily loads (TMDLs), as required in Section 303(d) of the Clean Water Act (CWA 4045 C.F.R.§130.2(i)). Watershed plans also can be developed proactively even before monitoring has established that designated uses of water bodies are impaired [1]. TMDLs have been developed for both small impaired waterbodies as well as large priority waterbodies (e.g., Gulf of Mexico and Chesapeake Bay). As climate changes, there is growing concern that TMDLs based on historical climate may not be achieved [2–5], either because of increased pollutant loadings with increased precipitation and runoff [2–4], or because of reduced effectiveness of best management practices (BMPs; [6–10]). In a few cases, climate change scenarios have been incorporated proactively into TMDL development [11]. Recently, the states implementing TMDLs for the Chesapeake Bay watershed have agreed to consider the effect of climate change on hydrologic conditions in the watershed in amending their Tier III Watershed Implementation Plans [12]. In other cases, litigation has forced states to update TMDLs to incorporate climate change impacts [5]. TMDLs are required to incorporate a margin of safety (MOS) in loading targets to account for uncertainties such as the effect of varying climate in supporting analyses, but rigorous methods for developing MOS values are rarely implemented [13].

City, county, and tribal planners and watershed managers often use models, simulations, and scenarios to consider different conditions, methods, and strategies for local and regional water management. There are several tools associated with existing watershed models that facilitate evaluating the effects of climate change on hydrology and water quality, such as:

- the Storm Water Management Model Climate Assessment Tool (SWMM-CAT; [14]);
- BASINS (Better Assessment Science Integrating Point and Nonpoint Sources)–HSPF (Hydrological Simulation Program–FORTRAN)–CAT (Climate Assessment Tool) modeling system [15];
- Climate Model Data for Hydrologic Modeling (CMhyd) tool for preparing bias-corrected climate inputs [16]; and
- the Hydrologic Comparison Assessment Module (HCAM; [17]) for the United States Environmental Protection Agency’s (US EPA) Watershed Management Optimization Support Tool (WMOST)).

Likewise, there are optimization tools designed to find the most cost-effective solutions to meet hydrology and/or pollutant loading targets under current climate conditions, generally focused on either urban or agricultural area-dominated watersheds [18–20]. However, there are relatively few applications of optimization programs to find least-cost solutions under climate change [21,22]. Tools to facilitate optimization of practices to meet TMDLs are needed to ensure investments in water quality protection are effective and cost efficient.

The goal of this project was to demonstrate the application of a novel suite of optimization tools developed by the US EPA to determine the least-cost management solution to meet sediment and phosphorus TMDL loading targets in a management area for both current and projected future climate conditions. This study provides a unique approach to simultaneously optimizing both urban and agricultural management solutions to meet loading targets under both historic and future climate scenarios, and incorporating uncertainty in future scenarios. Local stakeholders and the US EPA partnered in applying these tools to the Upper Soldier Creek (USC) subwatershed, a mixed land-use area, of the Middle Kansas watershed in Kansas [23]. The primary tool applied is the US EPA’s WMOST (www.epa.gov/hydrowq/wmost, accessed on 25 July 2025) [24]. WMOST is designed to support integrated water management, and is supplemented by a set of utility programs that support generating input files for WMOST for a range of future climate change scenarios. These utilities include:

1. Locating and Selecting Scenarios Online (LASSO), which summarizes key metrics from climate change scenarios [25];
2. HCAM, which facilitates Storm Water Management Model (SWMM) model simulations of urban stormwater BMP implementations under climate scenarios [26];
3. HCAM-R, which facilitates SWAT model simulations of agricultural conservation practice (ACP) implementations under climate scenarios [17]; and
4. the Climate Assessment Module Wrapper (CAM-WRAP), which combines outputs from the HCAM and HCAM-R applications to simulate or optimize solutions for a range of climate change scenarios [27].

## 2. Materials and Methods

### 2.1. Study Watershed

**Figure 1.**
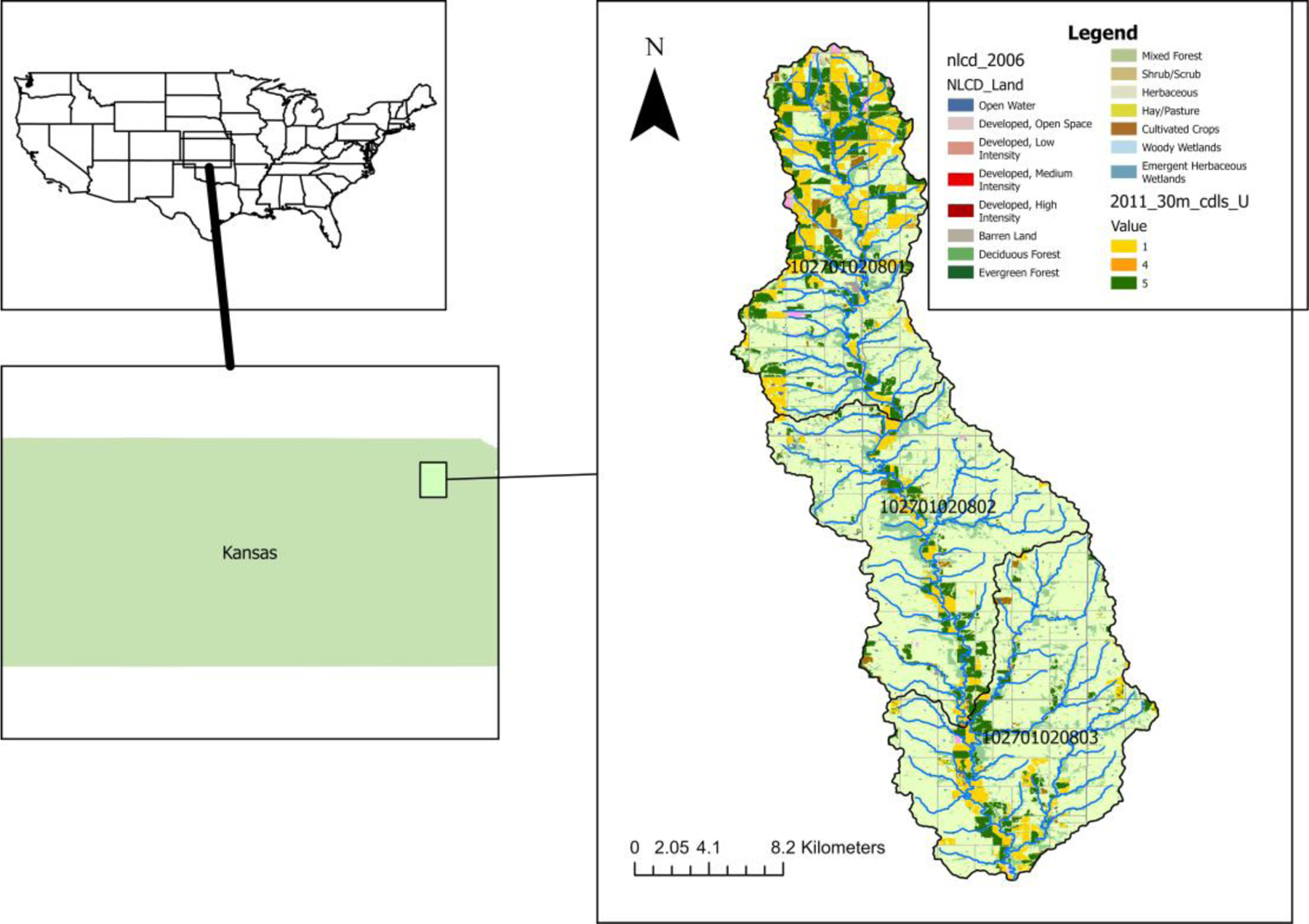
Upper Soldier Creek study area watershed in the state of Kansas, USA, with HUC12 boundaries and NLCD 2006 land use.

The 363 km^2^ study watershed representing USC is comprised of three 12-digit Hydrologic Units (HUCs) at the headwaters of the Middle Kansas River, Kansas: 102701020801, 102701020802, and 102701020803 (Figure 1). Based on the 2006 National Land Cover Dataset (NLCD) [28] used as the default for the modelling system (below), the USC subwatershed is primarily agricultural, with 64.3% hay/pasture/herbaceous, 20.0% cropland, 11.6% woodland, and only 3.5% low-density or open space developed land. Land cover within a 30 m (100 ft) buffer of rivers and streams within the watershed is half forested (49.4%), with lower cropland (12.7%) and hay/pasture/herbaceous coverage (33.1%) than the overall watershed. The USC watershed contains the Prairie Band Pottawatomie Nation reservation and the community of Soldier, both of which contribute permitted treated wastewater effluent discharges of sediment, nitrogen (N), and phosphorus (P). Point source contributions were included in calculations but represent a relatively small fraction of flows and pollutant loads. The watershed contains two small wastewater treatment plants with a combined operating capacity of 299 m^3^ day^*−*1^ (0.122 cfs) and permitted annual load of 373.3 kg (822.9 lb) total P per year. Although these additions to the stream were considered in calculations, optimizations focused on management practices for the dominant nonpoint sources instead of wastewater treatment plant upgrades.

The USC watershed is subject to a high priority TMDL for biology/sediment. The designated endpoint for the Biology TMDL is based on biological effects of suspended solids added to surface waters by artificial sources, with targets set to avoid impacts related to “the behavior, reproduction, physical habitat or other factors related to the survival and propagation of aquatic or semi-aquatic or terrestrial wildlife (KAR 28-16-28e(c)(2)(B))” [29]. Kansas Department of Health and the Environment (KDHE) established a target of average total suspended solids (TSS) levels below 100 mg/L at the United States Geological Survey (USGS) gauging station at Delia in USC, for flows less than 28.3 m^3^ s^*−*1^ (1000 cfs) (the 2% exceedance flow). That particular threshold was chosen because at levels below 100 mg/L of TSS, phosphorus and fecal coliform levels are also low.

We selected initial targets for the optimization based on the TMDL documented in the Middle Kansas Watershed Restoration Protection Strategy (WRAPS) 9 Element Plan Overview [23] (Table 1). The TMDL called for a 66% reduction from current values in annual TSS loads in the upper three subbasins of Soldier Creek (102701020801, 102701020802, and 102701020803). Current annual estimated sediment loads for nonpoint sources at the time of the WRAPS report were 25,310 mT (27,900 tons) TSS annually. The recommended loadings for the watershed were set at 8618 mT (9500 tons) TSS, calling for a reduction of 16,692 mT (18,400 tons) TSS in the annual loading (66%) to meet the sediment TMDL. This set the target for the optimization model at 8618 mT (9500 tons) of TSS per year.

The WRAPS document also specified imposing a TMDL for bacteria in the Upper Soldier Creek with 48% or greater annual load reductions. However, because of data scarcity and cost of bacterial monitoring, WRAPS authors determined that total phosphorus (TP) reductions could be used as a surrogate for bacterial load reductions. The WRAPS documents advise an annual phosphorus reduction of 488 kg (1076 lbs) over a 20-year period, with the goal of an annual reduction of 9764 kg (21,526 lbs) of phosphorus at the conclusion of the 20-year BMP implementation timeframe. Because no TMDL was imposed for TP, we used the annual load estimate for TP from the regional SPAtially Referenced Regression On Watershed (SPARROW) model for the Midwest (54,204 kg/yr or 119,500 lbs/yr) [30] and applied the reduction goals from the WRAPS document, thus giving an annual load target of (40,585 kg/yr or 89,474 lbs/yr).

Historic sources of both sediment and total phosphorus in USC include inputs from cropland and rangeland, inputs from cattle grazing near and within the stream corridor, and severe bank erosion from extreme events and from channel bed degradation progressing upstream from channelized areas downstream of the watershed [23,31,32]. Kansas stakeholders identified 9.4 km (30,919 ft) of hotspot channel bank erosion in USC in need of restoration, mostly in areas of row crop or sparse riparian vegetation [23]. The largest sediment yields in the Kansas River basin occur in the Dissected Till Plains of northeastern Kansas, where streams such as Soldier Creek incise the poorly consolidated glacial deposits, thus increasing average basin slope [33].

### 2.2. Overall Modelling and Optimization Approach

**Figure 2.**
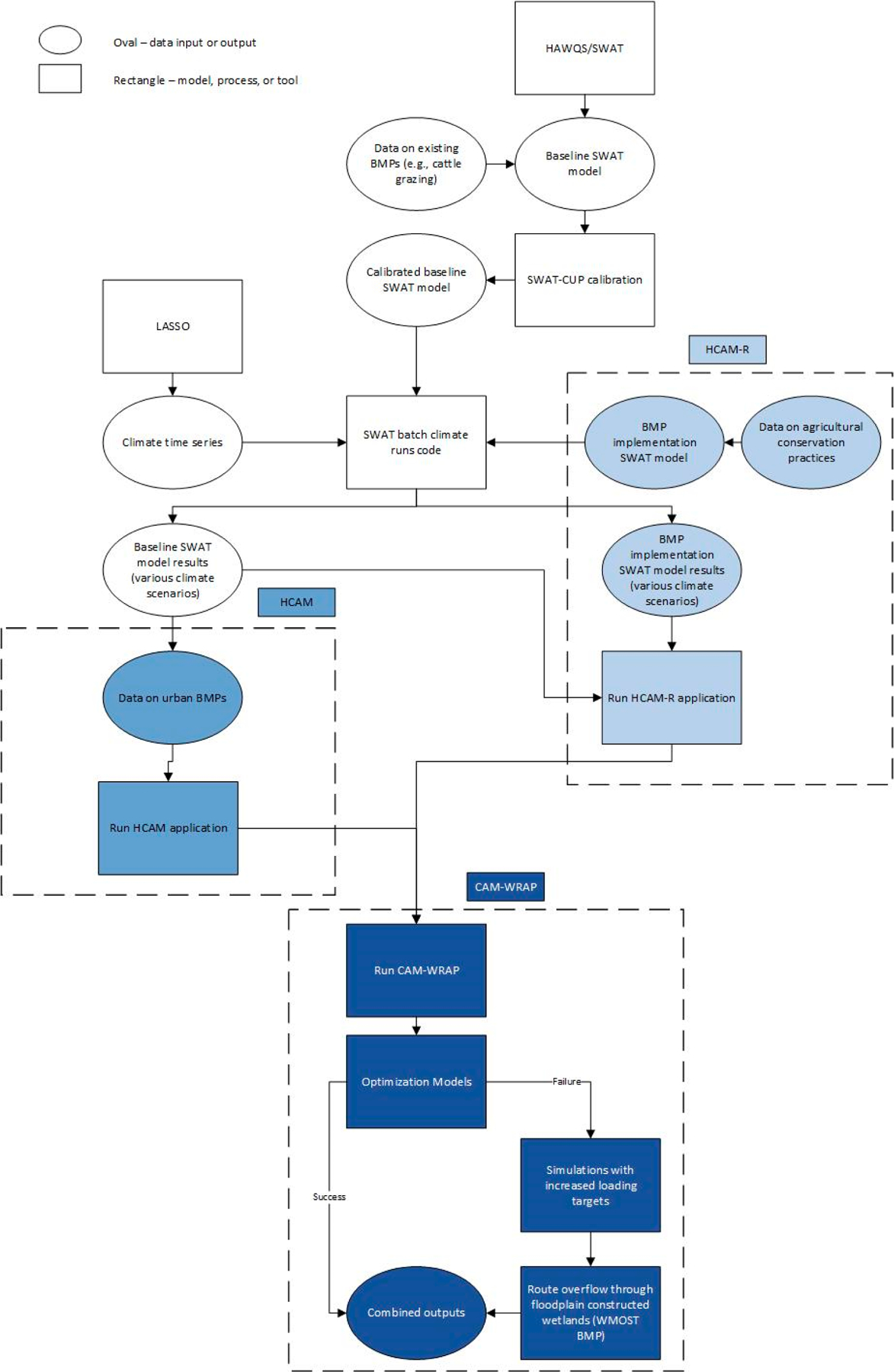
Flowchart of tool and model applications to determine the combination of least-cost management practices to achieve loading targets for Upper Soldier Creek watershed.

We determined least-cost management solutions to achieve TSS and TP loading targets for USC for current and future climate scenarios through the WMOST decision support tool and associated utilities (Figure 2). Based on user input, WMOST describes an optimization problem in a format that can be solved by optimization programs on the Network-Enabled Optimization System (NEOS) server [34], enabling users to find the least-cost solution to meet flow, loading, and/or concentration targets. WMOST requires inputs of daily hydrologic (runoff, recharge) and pollutant loading (runoff, recharge) time series by hydrologic response unit (HRU) to represent base conditions before new management options are implemented. WMOST also represents transfers between multiple water resource components, e.g., water consumption from groundwater or surface water, to wastewater, to septic or wastewater treatment plants, with discharge to groundwater or surface waters. Costs are entered to allow WMOST to allocate drinking water withdrawals between surface water, groundwater, and interbasin transfers to meet consumptive + nonconsumptive consumer demand, and wastewater discharge is estimated by WMOST based on consumptive use. In WMOST, aggregate flows, loads or concentrations are compared between baseline and “managed sets”, the latter representing time series after management practices are implemented. To define the base time series, we developed a watershed model for the case study area using the Soil and Water Assessment Tool (SWAT) supported by US EPA’s Hydrologic and Water Quality System (HAWQSv1) [35]. We simplified the hundreds of HRUs produced by HAWQS by aggregating down to seven categories for use in WMOST: Hay, cropland, forested, rangeland, urban, wetland, and water.

We modeled potential reductions in loadings related to implementation of ACPs externally in SWAT, and reductions related to urban stormwater BMPs internally within WMOST, respectively. WMOST is coupled with US EPA’s SWMM [36] behind the scenes to simulate effects of stormwater BMPs on hydrology and loadings, and generates managed sets for urban HRUs reflecting reduced runoff and pollutant loads. We focused on structural stormwater BMPs designed to increase infiltration, minimize runoff and associated loadings, and attenuate peak flow events (See National Menu of Best Management Practices (BMPs) for Stormwater-Post-Construction|US EPA (https://www.epa.gov/npdes/national-menu-best-management-practices-bmps-stormwater-post-construction, accessed on 25 July 2025) for examples). During the optimization process, HRU areas (decision variables) are allocated to different baseline or management treatments to find the least-cost solution to meet loading or flow targets.

We first explored optimal least-cost solutions for years of average precipitation (2014), drought (2012), and wet (2015) years. We then used US EPA’s LASSO tool to estimate potential changes in precipitation and temperature for a suite of future climate scenarios (Supplemental Material S1). We applied HCAM/WMOST and HCAM-R/SWAT to model the effects of stormwater BMP and agricultural conservation practice implementation, respectively, and generate modified hydrology and loading time series associated with future climate change scenarios and management options. We combined the agricultural conservation practice implementation with urban BMP implementation in WMOST using CAM-WRAP. Finally, we used WMOST in optimization mode to find least-cost solutions. When no solutions were generated given specified targets, WMOST was run in simulation mode with successively increasing targets. In simulation mode, no flow, loading, or concentration targets are specified, but user-chosen BMPs are implemented. In optimization mode, targets are specified and WMOST will select the least-cost solution to meet specified targets.

### 2.3. Development of SWAT Model for Upper Soldier Creek

The WMOST application requires a series of hydrology and runoff/recharge loading daily and hourly (stormwater runoff only) time series both for baseline conditions (baseline set) and for each management practice of interest (managed sets). These time series were generated from SWAT model outputs after aggregating time series into a simplified set comprised of five HRUs: Rangeland, Pasture, Forested, Cropland, and Low-Density Development. Managed sets for most agricultural conservation practices were generated from SWAT model scenario runs, while managed sets for urban stormwater BMPs were generated within WMOST using the stormwater module.

We created a baseline SWAT model for 1995–2015 (including a two-year warm-up period) at the HUC12-scale using the HAWQS v1 system. We used default data included with the HAWQS v1 system, including land-cover derived from NLCD 2006 and the Cropland Data Layer (2011–2012) [37] and weather inputs from the HAWQS Parameter-elevation Regressions on Independent Slopes Model (PRISM) database [35]. We updated the SWAT model to include point source inputs from wastewater effluent that were derived from (a) the US EPA Enforcement and Compliance History Online (ECHO) database (https://echo.epa.gov/trends/loading-tool/get-data/watershed-statistics, accessed on 25 July 2025) for TSS and (b) the Hypoxia Task Force database for TP (https://echo.epa.gov/trends/loading-tool/hypoxia-task-force-nutrient-model, accessed on 25 July 2025).

### 2.4. WMOST Applications

#### 2.4.1. WMOST Calibrations

WMOST data sources and derivation of input parameters are described in Supplemental Material S3. WMOST inputs included hydrology and loading (runoff, recharge) time series, HRU areas, surface and groundwater withdrawals for drinking water and irrigation, flow or loading targets, groundwater volumes, initial groundwater concentrations, and BMP costs and efficiencies (if not already modeled within WMOST). Hydrology and loading time series were derived from SWAT model output after SWAT was rerun with the “SWAT for WMOST and Hydroprocessor” SWAT version 2012 available at: https://swat.tamu.edu/software/ (accessed on 25 July 2025). This SWAT version expands on the default SWAT executable by providing a new series of .hru files that reorganize HRU-level outputs for each subbasin (oput*subbasin#*.hru), making SWAT outputs more readily usable by WMOST.

To bracket variability in BMP performance under recent climate conditions, we examined the modelled time span (1995–2015) for a year with near average precipitation (2014, 829 mm or 33 in), low precipitation (2012, 608 mm or 24 in) and high precipitation (2015, 1169 mm or 46 in) for Soldier Creek. Following calibration of the SWAT model, WMOST baseline models for the representative dry, average precipitation, and wet years were independently calibrated for flow as needed at a daily time step, e.g., adjusting the groundwater recession coefficient and groundwater volume parameters as needed to improve the baseflow fit.

#### 2.4.2. Applying WMOST to Find Management Solutions

We used WMOST in simulation or optimization mode to find least-cost solutions to meet loading targets. Effects of various management practices are represented in WMOST by creating “managed” time series for hydrology and loading variables for each HRU. In simulation mode, no flow, loading, or concentration targets are specified, but user-chosen BMPs are implemented. While in optimization mode, WMOST will select the least-cost solution to meet specified targets. Optimization definition files are uploaded to the NEOS server [34] and the mixed integer nonlinearly constrained Bonmin optimizer [38] is applied within the server to find the least-cost solution. If the specified targets cannot be met through optimization of management practices, then we systematically increase loading targets until we find one that is feasible, or add additional management practices for evaluation. Optimized solutions under current climate can be tested for feasibility under future climate scenarios by using WMOST in simulation mode with fixed BMP implementations.

### 2.5. Modeled BMPs and Agricultural Conservation Practices (ACPs)

We evaluated the effect of urban stormwater BMPs in WMOST. The stormwater module in WMOST is coupled with US EPA’s SWMM model in the background. We used default WMOST costs for urban BMPs, derived from the US EPA Region 1 Opti-Tool software v.1 [39].

We developed scenarios evaluating the effect of ACP implementation to generate “managed” time series for hydrology and sediment/nutrient loads. This was accomplished by modifying SWAT .mgt and .ops input files, generally following guidance in Waidler et al. [40]; See Supplemental Material S4 for details). The following agricultural conservation practices were simulated:

- No Till,
- Contouring,
- Contouring with Grassed Waterways,
- Terracing,
- Contouring with No Till,
- Terracing with No Till, and
- Vegetated Filter Strips

In addition, we simulated the addition of prairie pothole wetlands to capture excess runoff.

To evaluate the effect of cattle restrictions from streams, estimates of TP loads deposited directly into the stream by grazing cattle were added to the rangeland HRU. Then the cattle restriction ACP for rangeland was implemented as a “direct reduction BMP” in WMOST under the water quality (WQ) BMPs option (Supplemental Materials S2).

Constructed wetlands for agriculture were first simulated in SWAT as the addition of pothole wetlands, but pothole wetlands are implemented as permanent features within SWAT and restrict flow back to the main channel except during periods of high runoff when storage volumes are exceeded. This caused problems with maintaining positive stream discharge values. Thus, we also evaluated constructed wetlands for cropland using this BMP option in the WMOST stormwater module. The WMOST stormwater module can simulate either urban constructed wetlands using SWMM or constructed wetlands in agricultural settings (not linked with SWMM). However, this option could not be used in climate change scenarios using SWMM via HCAM (see below), which only handles urban BMPs. Ultimately, we simulated the use of off-channel constructed wetlands using the reservoir feature in WMOST as part of a two-tier approach (see below) that allowed excess (overbank) flows from the stream channel to be handled after upland BMPs were applied.

#### Riparian and In-Channel BMPs

Although WMOST has the capability to model the percent reduction (trapping) of sediment and nutrients from upstream sources by riparian buffers, these potential reductions are relatively small compared to the potential reduction of sediment and phosphorus inputs to USC from bank erosion as the result of bank stabilization conferred by forested vegetation [41]. Thus, we chose to model the stabilization function of riparian zones as a bank stabilization option in WMOST rather than as a reduction of upland inputs through the riparian module. We modeled a joint direct reduction BMP in WMOST as a combination of riparian restoration plus implementation of a two-stage channel design. The two-stage channel design has been used in agricultural settings in cases of significant channel incision where the stream has been disconnected from the historic floodplain, and provides a mini-floodplain that provides friction and improved settling of sediment with associated nutrients during events [42,43]. We also included a temporary bank stabilization option that can be implemented before riparian vegetation can be established. See Supplemental Materials S5 for details on parameterization of costs and efficiencies.

### 2.6. Current Climate

#### 2.6.1. Evaluating Upland, Riparian, and Channel Management Solutions Under Current Climate Conditions

We selected recent years for analysis to be representative of average precipitation (2014), relatively dry (2012), and relatively wet years (2015). To limit the number of management practices assessed in WMOST optimizations, we pre-screened ACPs to be included by comparing managed set loads with baseline loads and normalizing load reductions by practice costs to calculate a cost per mass of pollutant removal. WMOST prepares input files for optimization runs on NEOS (https://neos-server.org/neos/, accessed on 25 July 2025) [34], a publicly available server hosting a variety of optimization algorithms for public use. NEOS developers impose time limits on optimization problems to ensure that users do not tie up the resources with excessively long runs, so it is helpful to limit the number of managed sets in an optimization run to avoid solution timeouts.

Optimizations were set up for each year (2012, 2014, 2015) by WQ parameter combination using Type II validation files (see Supplemental Materials S2) as a starting point (Supplemental Materials S6). Optimizations included those ACPs showing overall load reductions for a given parameter plus the following options: biofiltration with underdrain and wet ponds, and the cattle-restriction BMP (stream fencing plus off-channel watering sources) as a direct reduction BMP. Targets for this set of runs were generated based on the 66% reductions in loads required by the Middle Kansas WRAPS, adjusted for the proportion of the load assigned to upland sources in the WRAPS document. WMOST provided least-cost solutions summarized by HRUs at the watershed scale.

We addressed streambank sources in a second set of optimizations including bank stabilization (physical + riparian restoration) and two-stage channel designs, and also via flow-based targets to manage sediment loads, the latter using methods developed by the Maryland Department of Environment [44,45]. When targets could not be met, WMOST was run in simulation mode starting with a much larger target and then with sequentially decreasing target load reductions to find the maximum load reductions achievable.

#### 2.6.2. Addition of Off-Channel Wetland Routing for Extreme Events in Current Climate Scenarios

Due to challenges in meeting the sediment loading target, TSS concentration target, and flow-based target to meet TSS target loads during high-flow events, we implemented a scenario to simulate the routing of excess flows (>28.3 m^3^ s^*−*1^ or 1000 cfs) to an off-channel treatment wetland, similar to the strategy implemented with the DesPlaines wetlands pilot [46]. The off-channel wetland was modeled as a reservoir in WMOST, with an initial volume of zero and costs for adding volume to force the model to find the optimal storage size to handle events over time. This is analogous to modifications to SWAT previously adopted to simulate floodplain wetlands [47]. Normally, the WMOST model can only simulate a fixed set of management practices during both baseflow and event periods, i.e., without switching management strategies from one time period to the next, so we implemented this strategy in stages, then combined results to represent the combined water quality results downstream of a return channel. We conducted a series of simulations with gradually increasing annual load targets to determine the minimum annual load achievable for 2014 with upland BMPs, starting with the original annual load target of nearly 12 million kg (over 26 million lbs) TSS/yr. The minimum annual load achievable was established as 64,775,808 kg (142,806,211 lbs) TSS/yr. WMOST was then run in hydrology-only mode for routing excess flows (>28.3 m^3^ s^*−*1^ or 1000 cfs) to a reservoir (proxy for the off-channel wetland), allowing the model to optimize added reservoir volume to minimize cost. Optimization/simulation of operations of the off-channel wetland (modelled as a pseudo-reservoir) focused on managing peak flows to minimize loadings from bank erosion based on flow-TSS relationships and thus did not take into account seasonal dynamics in nutrient processing that would take place in an actual wetland system.

We used a maximum surface water outflow from the reservoir of 4289.58 m^3^ s^*−*1^ (151,485.06 cfs) to limit what was returning to the stream reach. We derived this value from the maximum daily loading target (62,638 kg or 138,093 lbs TSS/day) and the established relationship between flow and TSS loads. The maximum daily loading target was derived using the methods of Maryland Dept of Environment [48] (MDE pp C-4 to C-5) incorporated into an SAS 9.4 program. Constructed off-channel wetland capitol and operation and maintenance (O&M) costs were derived from Kadlec and Hey [49] and Mitsch et al. [50], and adjusted for volume and volume pumped, respectively. Capital costs were estimated at ~$560,000 (the lesser of the two sources) and O&M costs for pump operations at $0.0001/MG. We estimated net flows and loadings downstream of the reservoir/off-channel wetland by combining (1) 2014 flows and loads from the original WMOST optimization based on upland BMPs, capped at flows of 12 m^3^ s^*−*1^ (410 cfs), with (2) the output from excess flows routed through the off-channel wetland/reservoir. TSS concentrations in reservoir outputs were estimated from the established TSS-flow relationship.

### 2.7. Evaluating Management Solutions Under Future Climate Conditions

#### 2.7.1. Selection and Generation of Future Climate Scenarios with LASSO

Using US EPA’s LASSO tool [25], we summarized 32 climate change projections to evaluate potential seasonal and annual changes in air temperature and precipitation between the baseline period of 1987–2016 and the simulated future period of 2021–2050 (centered on a 20-year implementation endpoint of 2036) using the Localized Constructed Analogs (LOCA) data source under the Representative Concentration Pathway (RCP) 4.5 emissions scenario (Supplemental Material S1). The LOCA projections are statistically downscaled Coupled Model Intercomparison Project Phase 5 (CMIP5) climate simulations at 1/16*◦* spatial resolution. The RCP4.5 emissions scenario assumes that global annual greenhouse gas emissions peak around the year 2040 and then gradually decline with worldwide adoption of some emissions mitigation policies, and assumes an average radiative forcing value of 4.5 W/m^2^ in the year 2100. From these results, we plotted bounding envelopes on scatterplots of temperature versus precipitation change to identify the full range of projected climate change metrics, and narrowed the number of climate change scenarios for WMOST optimizations/simulations from 32 down to 14, representing the diversity of future climate projections (Supplemental Material S1 and S5).

Simply applying annual delta change factors from climate change scenarios to original weather time series can obscure seasonal variations in temperature change and changes in the frequency distribution of precipitation events. Therefore, we calculated monthly change deltas by comparing climate change scenario time series with baseline observed temperatures, then applied average change values to the original air temperature time series. For precipitation, we applied methods outlined in Maimone et al. [51] to generate future climate scenarios with the correct frequency of different storm intensity events. Precipitation percentiles are calculated by season for future climate change scenarios (average across subbasins). Delta change factors are calculated for matching with original precipitation events, which are adjusted accordingly (Supplemental Material S7).

#### 2.7.2. Simulation Runs Under Future Climate with Current Maximum-Load-Reduction Management Practices

Due to difficulties in achieving loading targets for TP and TSS under current climate conditions, we chose to run WMOST in simulation mode (without target loads or concentrations), rather than in optimization mode, for the 14 selected climate change scenarios. We implemented the BMPs that achieved the loading target for TP for the normal precipitation year (2014), then assessed the frequency of exceedances of daily and annual loading targets under future scenarios. Based on this initial exercise, the four most optimistic climate change scenarios (lowest incidence of exceedances) were selected for optimization through addition of in-channel and riparian stabilization practices to maximize the chance of finding a solution.

## 3. Results

### 3.1. SWAT Model Calibration

Acceptable calibration statistic thresholds for Nash-Sutcliffe Efficiency (NSE) and root mean square error (RMSE) have been published by Moriasi [52], but they refer to calibration of monthly values, so should be considered conservative in judging outputs from our daily SWAT model. The Kling–Gupta Efficiency (KGE) statistic can range from negative infinity to 1 (perfect agreement of mean, variability, and timing of observed values) [53]. Percent bias for flow was very good for subbasin 1 (USC headwaters) and satisfactory for subbasin 3 (USC mouth), while the KGE) and RMSE-observations standard deviation ratio (RSR) were satisfactory for all three (Table 2). TSS loading from subbasin 3 had a marginal percent bias (56.5 > 55% threshold for monthly values) but unsatisfactory NSE and KGE values. However, the NSE value still exceeded the baseline (*−*0.41) expected when results are no better than random [54], and fell within the range reported in the literature for daily calibrations (*−*3.51–0.23, median = *−*1.64). The TP r^2^ value was reasonable (0.76), but TP daily calibration bias at the mouth of USC was unsatisfactory compared with monthly thresholds (abs(*−*58.1) > 55%), and NSE was unsatisfactory. No separate SWAT model validations were run due to the inadequate number of observed values for TP, TSS, and total nitrogen (TN). The fit was improved during WMOST calibrations (see below) by increasing groundwater concentrations (default concentration of zero in SWAT) and adjusting point source inputs to partially correct for wastewater treatment plant (WWTP) measurement errors.

### 3.2. WMOST Model Calibration

Type II model calibrations for 2014 with WMOST were excellent for hydrology, with a daily NSE of 0.801, r^2^ value of 0.836, and median bias of 16.76. Calibrations for both TP and TSS were severely constrained by a lack of supporting water quality data after 2007, so we made comparisons of Type II WMOST 2012 model calibrations with results of our SWAT model and the 2012 USGS SPARROW model predictions. WMOST Type II model calibrations yielded results of 3314 kg P/yr (7306 lbs P/yr), intermediate between SWAT (2323 kg or 5121 lbs P/yr) and SPARROW results (16,181 kg or 35,673 lbs P/yr). SWAT and SPARROW modeled TSS loads for 2012 were similar, at 31,064 mT TSS/yr (34,242 tons/yr) for SWAT and 38,776 mT TSS/yr (42,743 tons/yr) for the SPARROW model, differing by 19.9%. WMOST estimates with (199,860 mT or 220,308 tons TSS/yr) and without (28.0 mT or 30.9 tons TSS/yr) bank erosion bracketed SWAT and SPARROW model results.

### 3.3. Least-Cost Optimization to Meet Upland TP and TSS Loading Targets Under Current Climate Conditions

The upland TP loading target for the dry year 2012 could be met with existing BMPs (obtained from KDHE), with no added costs beyond the base level of $1.107 million required to support existing water resources infrastructure, because the target was based on reductions from loads experienced in an average precipitation year (2014). For 2014 (average precipitation) conditions, the upland TP loading target could be met at least cost ($3.224 million) through an addition of grassed swales with underdrain for urban area runoff (3.1 km^2^ or 755 acres treated for $1.059 million) and contouring for agricultural area runoff (full 71.3 km^2^ or 17,617 acres of agricultural area treated for $105.7K). For the wet year of 2015, the upland TP loading target could be met at least cost with the same treatment for agricultural area and with a switch to sand filter with underdrains treating 8.9 km^2^ (2210 acres) and a lower total management cost of $3.01 million. A storage-based solution could be utilized with wet ponds treating 9.9 km^2^ (2435 acres) of developed area and a total management cost of $3.19 million. However, with the added storage, a groundwater supplement would be required to maintain baseflows. For TSS, upland loading targets could not be met for any of the years tested, dry, wet, or average.

### 3.4. Staged Optimizations to Meet Combined Upland and Bank Erosion Loading Targets for TSS

**Figure 3.**
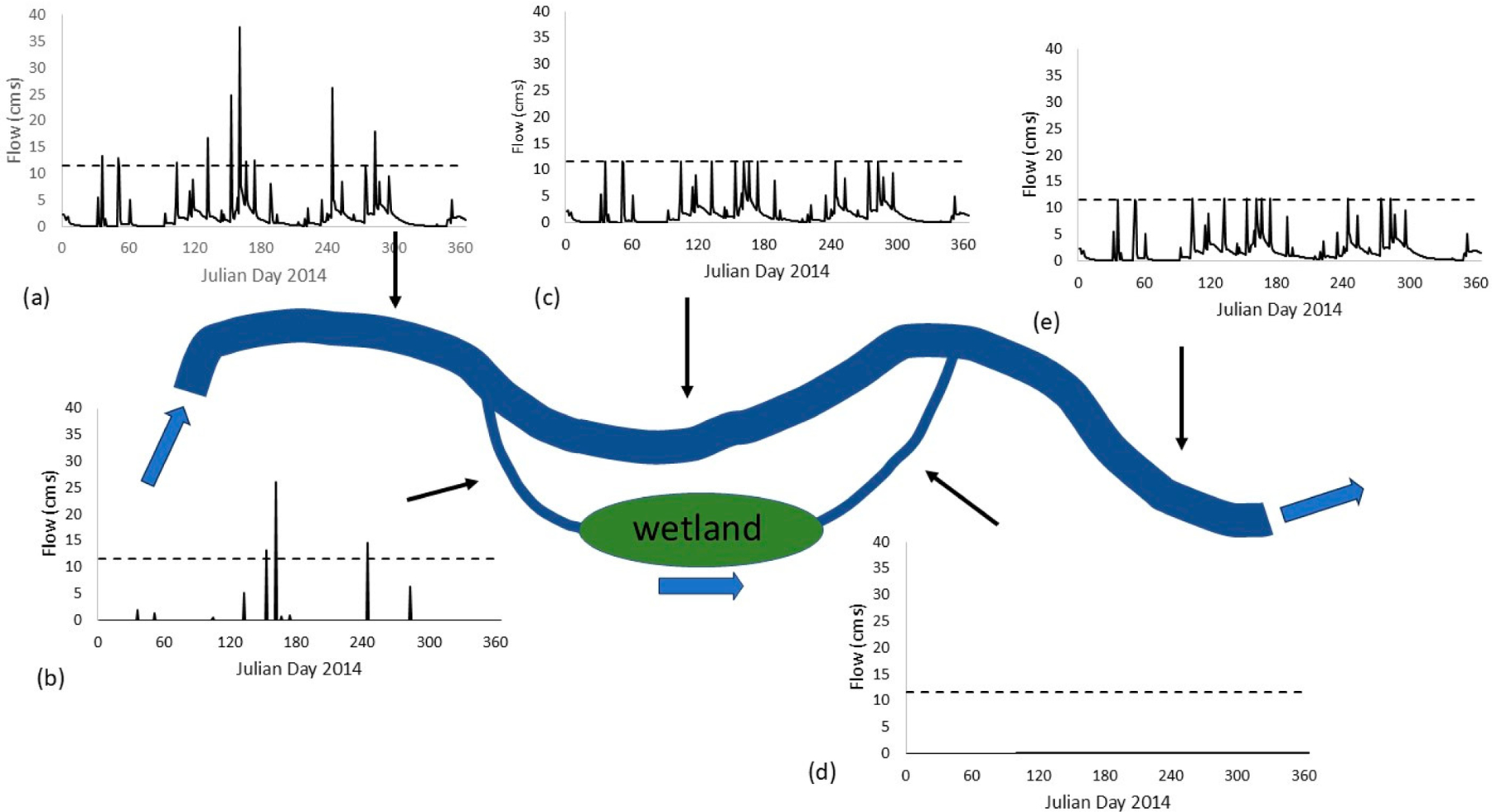
Attenuation of flows through off-channel wetland. Flows from 2014 (**a**) exiting from upland BMP scenario with partial implementations of cattle exclusion, grassed swales (for urban runoff), terracing/no till treatment of cropland, and stream stabilization/riparian restoration are partitioned into (**b**) excess flows (above target flow value) and (**c**) below target value. Excess flows are routed through an off-channel wetland with (**d**) significantly reduced peaks. Combined flows (**e**) where the diversion joins the main channel meet the target flow, with the exception of one slight exceedance. The target flow value is indicated with a dashed line.

As described above, using the outputs from the upland BMPs model, we developed the input time series for the extreme flows model in WMOST, which routed “excess” flows—-the difference between treated flows and target flow values—-through a simulated off-channel wetland (Supplemental Materials S8). For 2014, different results for optimizing upland BMPs were obtained based on whether the results included terracing + no till versus contouring. The former solution included treatment of 1.1 km^2^ (277 acres) with terracing + no till, treatment of 13.3 km^2^ (3297 acres) of developed area with grassed swales with drains, 3328 m (10,920 ft) of bank stabilization/riparian restoration, as well as cattle exclusion. This solution was more expensive ($17M versus $2.4M), but it included bank stabilization/riparian restoration, while the contouring solution did not. We chose to model the more expensive case because it would allow us to use the post-BMP discharge—TSS load relationship (assuming bank erosion would be controlled even after routing flows from the off-channel wetland back to the main channel) to estimate target flows needed. The predicted storage needs for the off-channel wetland based on 2014 conditions were 3421,255 m^3^ (903.8 MG) with an added cost of $34M, for a total of $51M (upland BMPs + off-channel wetland; Figure 3).

### 3.5. Least-Cost Optimization to Meet TP and TSS Upland Loading Targets Under Future Climate Conditions

#### 3.5.1. Least-Cost Optimization to Meet TP Loading Targets Under Future Climate Conditions

**Figure 4.**
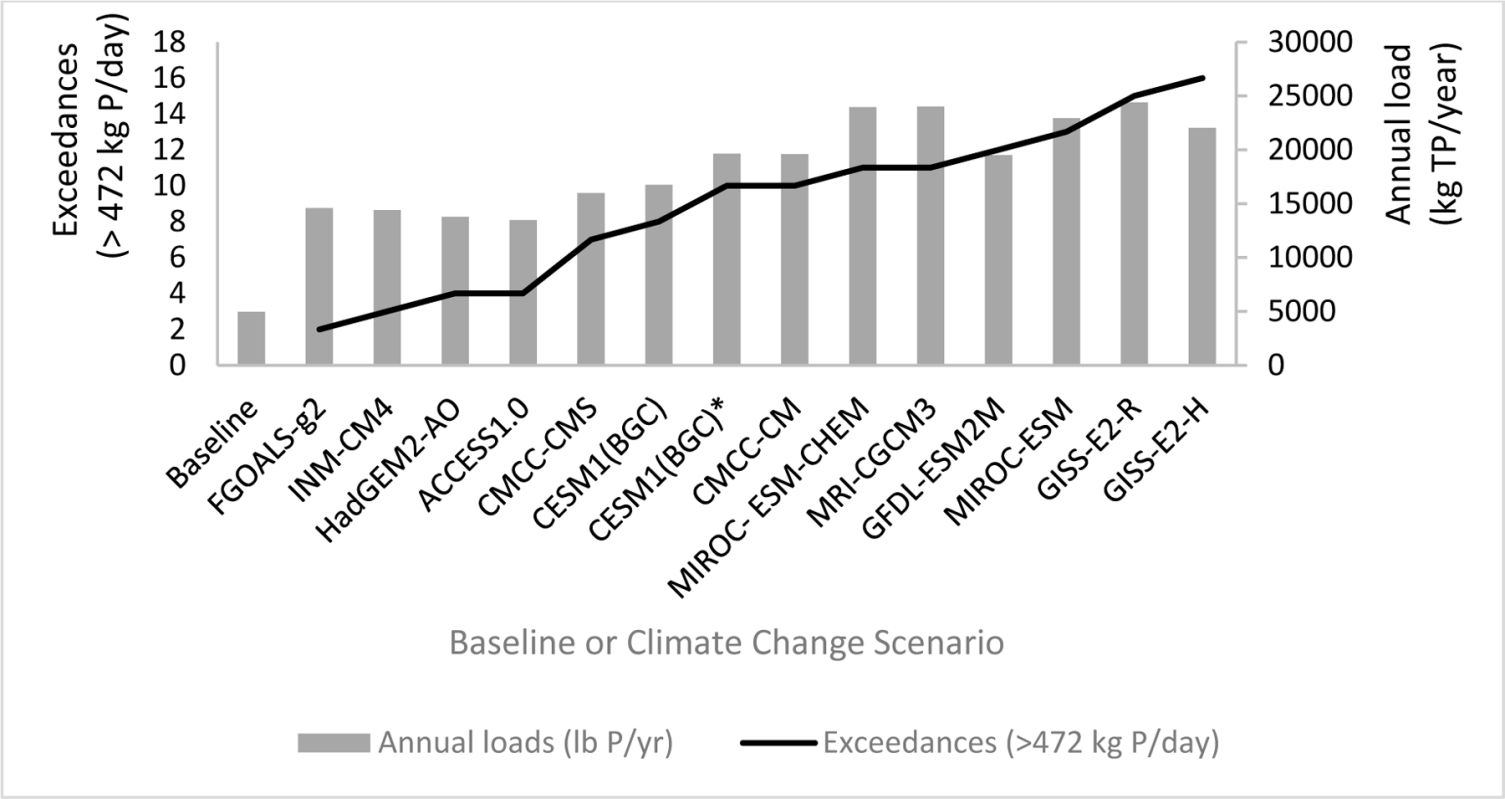
Climate Scenario Summary. RCP4.5 scenarios for 2021–2050 timeframe as compared to 2014 baseline. Simulations were run with an initial groundwater concentration of zero parts per million (ppm) TP except as indicated by * (0.04 ppm TP). Annual total TP loads (lbs TP/yr) and number of exceedances of annual daily load target (>471.6 kg or 1039.6 lb TP/day) at mouth of USC are shown for each climate simulation.

We first compared model results against the daily and annual loading targets: 471.6 kg/day (1039.6 lbs/day) and 4950 kg/yr (10,913 lbs/yr), respectively. All climate scenario model simulations resulted in at least one exceedance of the daily loading target, and all climate scenarios exceeded the annual loading target (see Figure 4). Daily and annual loading target exceedances are highlighted in red on the “Table_LSw1” tab of the ScenCompare results comparison file for reference (Supplemental Material S9).

**Figure 5.**
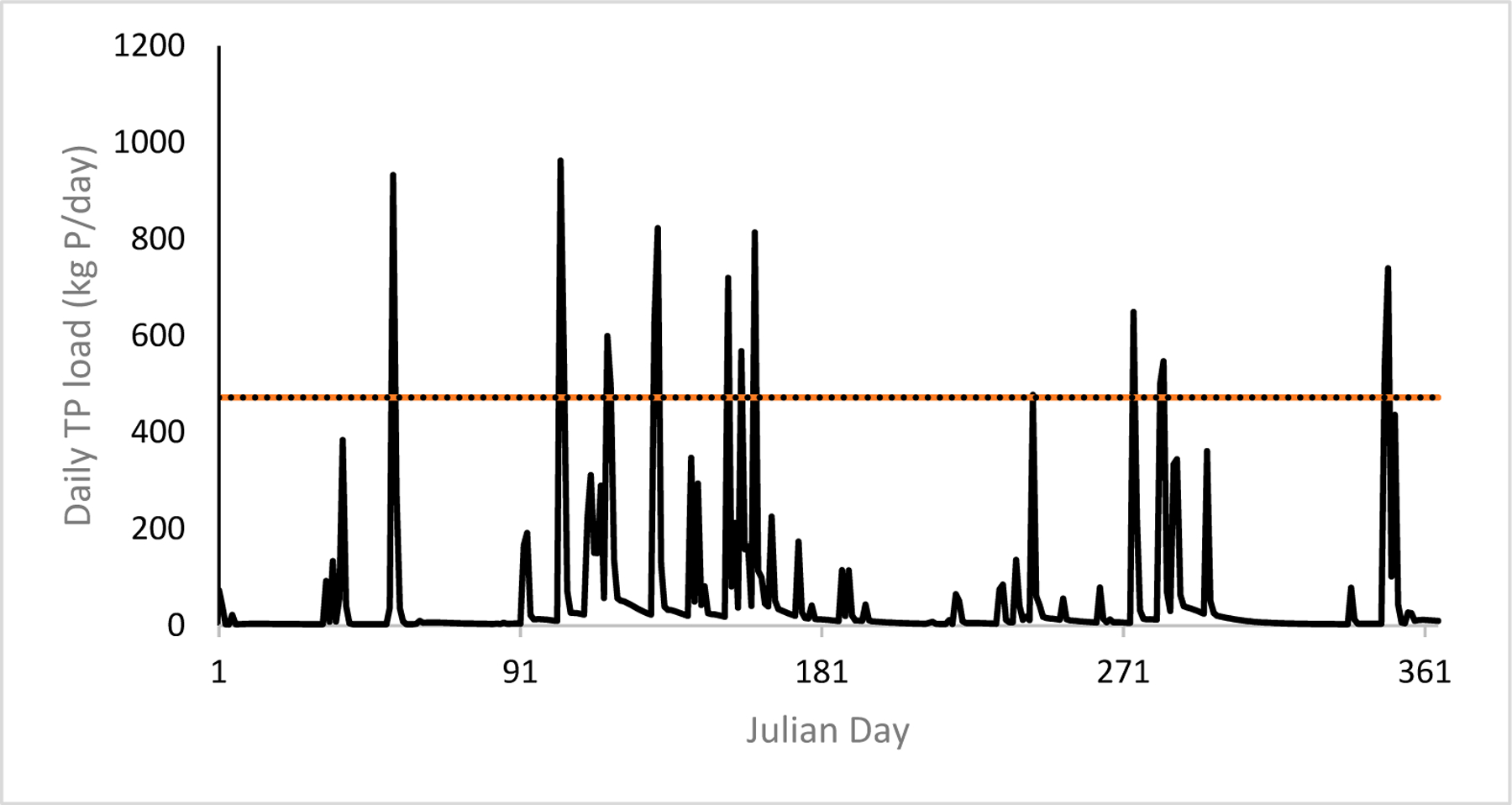
Predicted daily loads normalized for year of average precipitation adjusted for climate change (2021–2050) based on GISS-E2-H change scenario for RCP4.5 and implementation of least-cost optimized management solutions for 2014. Target load of 471.6 kg P/day is shown with dashed line to highlight projected exceedances.

The GISS-E2-H climate scenario model simulation results exceeded the daily loading target the most with 16 exceedances (Figure 5), while the FG FGOALS-g2 climate scenario model simulation results exceeded the daily loading target the least with two exceedances. Exceedances above the daily loading target were on average about 181 kg (400 lbs) greater, although some model results had daily loadings that were around 23,269 kg (51,300 lbs) over the target. The annual target was exceeded by every climate scenario, with the lowest total annual loadings of 13,446.10 kg (29,643.58 lbs) for climate scenario ACCESS 1.0 and the highest total annual loadings of 24,368.57 kg (53,723.51 lbs) for climate scenario GISS-E2-R. Overall, the annual exceedances ranged from triple to quintuple the target annual loading value. Given the difference between simulated loadings and the annual loading target, we moved forward with additional testing to meet the daily loading target only.

We proceeded with the optimization of the four models with fewer than five daily exceedances (FGOALS-g2, HadGEM2-AO, INM-CM4, and ACCESS1.0) using the daily loading target. Of these four models, only HadGEM2-AO successfully optimized through NEOS. This is likely because the total annual load for the WMOST simulation using the HadGEM2-AO climate scenario was the lowest of the four models. The optimized cost to meet the daily loading target under the HadGEM2-AO scenario is $2.16 million compared to the baseline scenario cost of $3.2 million. The HadGEM2-AO scenario model optimization cost is less than the baseline scenario cost because the baseline scenario included both daily and annual surface water loading targets, requiring the implementation of the more expensive grass swale management practice. Both scenarios had similar costs otherwise, including choosing a similar amount of land area to implement contouring practices. Examining the surface water loading between the two scenarios, the HadGEM2-AO scenario’s annual loading is 12,560.97 kg (27,692.19 lbs), which is 7610.91 kg (16,779.19 lbs) higher than the annual loading from the baseline scenario. The maximum daily loading rate in the optimized HadGEM2-AO scenario is 471.6 kg (1039.6 lbs) compared to the baseline scenario with a maximum daily loading rate of 408.56 kg (900.73 lbs). The average daily loading rate for the optimized HadGEM2-AO scenario is 34.41 kg (75.87 lbs) per day, while the rate is 13.5 kg (29.9 lbs) per day for the baseline scenario.

As described above, the contouring, extended dry detention basin, and grass swale BMPS did not reduce loads enough to meet the TMDL loading targets even across the four climate scenarios with the fewest exceedances. We conducted a subsequent analysis into the sources of phosphorus in the modeled area to determine why the loading targets could not be met. We found that over 60 percent of the phosphorus loads entering the stream came from range and hay land uses.

We implemented a streambank stabilization or streambank restoration BMP, as bank erosion is a large contributor of phosphorus and sediment loads in this watershed (Supplemental Materials S5). Since the removal rate depends on baseline loadings that differ across the climate scenarios, we tested two model scenarios: one using the average removal rate of TP across the 14 climate scenarios (2.55 *×* 10^*−*4^ kg or 5.63 *×* 10^*−*4^ lbs/ft/timestep) and one using the minimum removal rate of TP across the 14 climate scenarios (1.82 *×* 10^*−*4^ kg or 4.00 *×* 10^*−*4^ lbs/ft/timestep). None of the climate scenarios optimized with the daily loading target. The target daily load had to be increased 1.4 times to affect a solution with the FGOALS-g2 scenario, which had the lowest maximum daily load across climate models, but stream bank stabilization was not selected.

#### 3.5.2. Least-Cost Optimization to Meet Upland TSS Loading Targets Under Future Climate Conditions

Simulations were also run for TSS management to see if TP solutions would be effective for meeting TSS targets in future climates. Simulations for TSS management under future climate scenarios were based on the optimization for 5.8 times the original target, which incorporated contouring, urban BMPs, no agricultural constructed wetlands, and no bank stabilization to match selected options for TP management. Projected TSS loadings with the current TSS load reduction strategy implemented under climate change ranged from 2 to 5.5 times larger than baseline loadings for 2014 so did not meet the target. Although we were able to get an optimized solution to meet TSS load targets under the current 2014 climate scenario using bank stabilization options, the solution was much more expensive, so we did not repeat TSS simulations for future scenarios with bank stabilization.

## 4. Discussion

### 4.1. Utility of WMOST and Associated Utilities in Evaluating Least-Cost Management Options

We demonstrated the utility of the WMOST integrated modelling suite to identify what combinations of upland BMPs, riparian and in-channel management, and off-channel solutions are most cost-effective in treating upland and bank-erosion sources of TP and TSS under variable weather conditions. However, solutions that are successful and cost-effective under dry or average precipitation years, may be inadequate during wet years or under changing climate conditions, requiring that larger margins of safety be built into implementation plans. When targets cannot be met, then loading targets can be systematically increased to find the lowest target achievable.

### 4.2. Upland vs. Instream/Riparian Sources of TSS and TP

The relative effectiveness of BMPs focused on different landscape components (uplands, riparian zone, stream bank, channel) in reducing TSS and TP inputs to streams will depend on the predominant source of sediments and may vary with flow magnitude. Bank erosion inputs were estimated to be a significant source of sediment, and thus TP, in Upper Soldier Creek (Stakeholder Leadership Team 2011). Bank erosion is being recognized as the major source of sediment and phosphorus loads in many watersheds, including those in northeastern Kansas [55].

Overall, our optimizations focused on reducing sediment during peak flows rather than low to medium flows, and thus upland BMPs were found to be less successful at meeting targets. Factors controlling sediment loads can shift from percent agricultural land-use at low- or medium-frequency flows to soil erodibility and predominance of floodplain wetlands for extreme events, particularly for channels that have become incised [56], and thus different control strategies may be needed for different flow frequencies. In addition, agricultural practices have led to storage of fine legacy sediments within streams and floodplains, which can be readily mobilized during low flows [57]; thus, practices that increase sediment trapping within the channel, e.g., two-stage ditch solutions, may be more effective at minimizing effects of legacy sediments at low to medium flows than solely controlling sources in uplands.

### 4.3. Limitations on Representation of ACPs

Our ability to represent the pollutant removal effectiveness of some agricultural practices was restricted somewhat by a combination of factors: reliance on empirical estimates of riparian restoration effectiveness under historic hydrologic regimes, inability to model pollutant load reductions from agricultural lands in response to regenerative agriculture practices, and inability to model the effectiveness of grazing practices such as rotational grazing. Representation of riparian buffer performance has typically focused on enhancement of denitrification in saturated soils and on sediment trapping efficiency with removal of associated pollutants [58], and nutrient/removal efficiency estimates incorporated into watershed models and into the riparian buffer module of WMOST reflect these functions. However, the stabilization function of riparian vegetation in reducing bank erosion can be much more significant in the long run for systems with chronic bank erosion, particularly for reducing inputs of sediment and associated pollutants such as phosphorus. Thus, in modelling riparian functions within WMOST for this case study, we evaluated riparian restoration effectiveness based on empirical estimates of effects of riparian vegetation establishment on regional bank erosion rates. However, effects on bank erosion rates were measured under historic conditions and may not adequately represent effectiveness under current or future extreme events.

To deal with increased loadings associated with more frequent rain events and associated bank erosion, long-term regenerative agricultural practices may be needed to improve soil health (e.g., soil organic matter, macropores) that influences water retention in the uplands. Many traditional upland BMPs are designed to reduce soil erosion, trap sediment with vegetative filters, or increase uptake of nutrients in streamside buffers, but do not necessarily reduce the runoff volume as much as sediment inputs [59]. Estimated runoff volume and pollutant export based on SWAT is most sensitive to the curve number (CN) parameter, but suggested modifications to the CN for various agricultural conservation practices typically involve CN adjustment by only a few units [60]. Thus, SWAT-based simulations of the effectiveness of ACPs in the Susquehanna River Basin under climate change found that most ACPs do not appreciably change the water balance, although some reduce sediment and nitrogen export [61]. Greater improvements may be possible with regenerative agriculture practices that employ long-term application of conservation practices over a decade or more, with subsequent improvements of soil health, but more quantitative studies are needed to document long-term changes in soil properties [62,63]. The SWAT model defines HRUs on the basis of land-use, soil hydrologic group, and slope—of which, only land-use may vary with conservation practices. However, SWAT includes soil parameters such as organic matter, infiltration rate, and depth to impermeable layer, which influence water retention, and which could be updated in simulations of effects of regenerative agriculture if supporting data were available.

Inadequate information has been available to inform adjustments to the SWAT model to mimic effects of rotational grazing on soil health to enable these practices to be incorporated into optimization exercises, such as the one we conducted for USC. With respect to upland sources in the USC watershed, rangeland contributed the most to runoff, but only one ACP option was targeted to rangeland management, i.e., cattle exclusion from streams. While cattle exclusion reduces both riparian and direct instream inputs of manure, it does not affect the water balance and runoff volume from uplands with attendant effects on bank erosion. There are rangeland management practices associated with regenerative agriculture such as rotational grazing that can improve soil health (increasing soil organic matter with improved water retention and aggregate stability with reduced erosion) but only recently have studies been initiated to quantify their benefits [64]. Simulations of switching to rotational grazing from traditional agricultural practices has been associated with improved soil health and up to 96.0%, 89.4%, and 99.9% reductions in loads of nitrogen, phosphorus, and sediment, respectively, but results vary widely across farms [61].

### 4.4. Need for Staged BMP Strategies Under Climate Change in Semi-Arid Climates to Deal with Extreme Events

The increasing frequency and magnitude of extreme events requires that management strategies be able to moderate flow and pollutant loads over a wide range of conditions, but optimal strategies in years with extreme events may require unique strategies that are not effective or are counter-productive in years with low or average precipitation. In addition, there are limitations in both static optimization processes and in watershed models in simulating the dynamic wetland connections in the landscape that influence how water storage and pollutant removal efficiencies vary with climate regime.

Most optimization approaches for watershed management such as those used by WMOST are designed to find static management options that are chosen to be implemented for the full timeline of the model application. With this approach, we can optimize management options for relatively wet or dry years independently, but in most cases, we cannot readily simulate turning management options on and off or re-routing flows in response to extreme events. Reservoir management is the one exception, for which dynamic behavior can be implemented in WMOST via decision rules. In SWAT, there are additional limitations for representing wetland function under dynamic climate conditions. In SWAT, constructed agricultural wetlands can be modeled through the addition of prairie pothole wetland objects; however, in SWAT, these elements only release water to a downstream channel when they fill up and overflow. Thus, in dry years, modeled results show storage in the landscape theoretically restricting or eliminating flow in stream channels. Modelling dynamic connections to floodplain wetlands is also not available in the base SWAT model, although some specialized versions not yet publicly available have been designed for this purpose [65]. Dynamic connectivity with floodplain wetlands can also be more readily modeled in SWAT-Plus [66]. This limitation was particularly problematic for watershed management optimizations in semi-arid regions, such as USC, that are also subject to extreme events. Potential solutions to this limitation include:

- use of SWAT-Plus or a modified SWAT model that incorporates riparian wetland dynamics [67],
- application of a staged BMP approach that optimizes upland BMP selection for flows less than bankfull (or some other threshold) and independently routes overflows to an off-channel wetland or other control structure, or
- switching to a dynamic optimization modelling approach [68,69] or integrated modelling approach that incorporates submodels that incorporate near-channel processes [70,71].

### 4.5. Uncertainties in High Costs of Off-Channel Wetlands Storage and Opportunities for Reducing Net Costs via Co-Benefits

The accuracy of our cost estimates for the final tiered solution was constrained by limited data on actual costs of constructed off-channel wetlands and did not consider additional co-benefits that would reduce the net cost. There are relatively few estimates of the cost of constructing off-channel wetlands based on actual projects treating upstream watersheds, and costs [45,46] vary widely due in part to variation in costs of underlying land [67] and differences in implementation details [72]. Nietch et al. reported on estimated costs for potential wetland creation projects in the East Fork of the Little Miami River watershed, with a range of $300,900/km^2^ ($3009/ha) treated for floodplain wetlands, to $955,600/km^2^ ($9556/ha)–$1,214,900/km^2^ ($12,149/ha) for pump-and-treat off-channel wetlands, with even greater initial actual expenses of $37.5 million per km^2^ ($375,000/ha). Hansen et al. [67] applied modelling with multi-objective evolutionary optimization algorithms (MOEA) to a wide range of scenarios to evaluate costs of simultaneously reducing sediment and nitrogen in the Le Sueur River Basin (LSRB), a subwatershed of the Minnesota River basin. For $12M/yr ($4140/km^2^ or $41.40/ha crops/yr), a 77% reduction in sediment inputs and a near 100% reduction in nitrogen inputs could be achieved, with field-derived P reductions of 46–65%. Optimal control strategies were dominated by near-channel management strategies, such as fluvial wetlands, rather than upland management practices. Due to their greater cost-effectiveness, numerous small, shallow fluvial wetlands (0.02 km^2^ or 2.02 ha, average depth < 1.1m) were favored over fewer larger or deeper wetlands, such as those implemented in the DesPlaines and Miami River case studies that we based our cost estimates on.

The net cost of implementing off-channel wetland storage could be reduced by considering associated co-benefits such as reduction of flood risk, maintaining reservoir capacity by reducing sediment infill rates, and carbon sequestration benefits. The cost of removing just the average annual sediment load entering the 24 Federal reservoirs in Kansas has been estimated at $106 million/yr [73]. According to the First Street Foundation national flooding risk model [74], there are 4306 properties in downstream Topeka, KS, that have a greater-than 26% chance of being severely affected by flooding over the next 30 years. This represents 18% of all properties in Topeka (https://riskfactor.com/city/topeka-ks/2071000_fsid/flood, accessed on 25 July 2025). For reference, the flood of 1951 in northeastern KS produced damage costs exceeding 760 million dollars, which would be over 5 billion dollars today (https://www.weather.gov/top/1951_flood, accessed on 25 July 2025). A series of reservoirs and levees were constructed in response to the floods of 1951, but these tend to be located closer to population centers. Currently, reservoirs in Kansas provide multiple benefits (flood management, drinking water, recreational benefits), but are at risk of loss of capacity due to sediment infilling [73], and their functions are at risk due to increased incidences of harmful algal blooms fueled by excess nutrient inputs [75]. Their functional lives could be extended and health risks reduced by restoring storage capacity upstream, either through distributed wetlands or off-channel wetlands in the floodplain. The US EPA’s existing ScenCompare CoBenefits module could be updated to better incorporate the cost savings associated with these additional benefits.

## 5. Conclusions

Potential costs and extents of BMPs and ACPs designed to meet TP and TSS loading targets in Upper Soldier Creek varied significantly between years with relatively low (2012), average (2014), and above average (2015) precipitation (Table 3), with a tendency to fluctuate from infiltration-based to storage-based solutions as precipitation increased. While existing management practices were sufficient to meet TP loading targets in a dry year, practices costing 1–2 million dollars were required in years of average or above average precipitation. Due to the significant contributions of bank erosion to sediment inputs, meeting TSS target loads will be even more challenging, requiring a combination of upland, riparian, bank-stabilization, and off-channel storage to meet relaxed targets in an average precipitation year.

The integrated WMOST models were useful in evaluating potential successes and constraints of different suites of management practices involving upland BMPs, riparian and in-channel management, and staged solutions involving off-channel wetlands to treat overflows during extreme events. Traditional upland ACPs are constrained in their ability to limit loadings of sediment and associated pollutants, such as TP for systems like USC, which have loadings dominated by bank erosion. This is because upland ACPs are designed to trap sediment and nutrients, but not optimized to reduce runoff volume to the extent needed. It is possible that emerging long-term regenerative practices to manage both grazing practices and cropping systems will be more successful in managing agricultural runoff volumes to reduce the magnitude of extreme events, but more quantitative data are needed to parameterize existing models to reflect long-term changes in soil structure and composition. In addition, water storage-based solutions, such as distributed constructed wetlands or off-channel floodplain wetlands, are needed to deal with erosivity of excess flows associated with extreme events; the latter can be simulated in a two-stage optimization process, with the excess flows routed through an off-channel storage system. Our two-stage optimization process identified a solution to meet maximum daily flows for TSS for a year, with average precipitation that incorporated protections at all levels: uplands, riparian zones, channel, and stream banks.

## Supplementary Material

Supplement1 The following supporting information can be downloaded at: https://www.mdpi.com/article/10.3390/w17152265/s1, Figure S1a–e in Supplemental Materials S1: Climate change scenario definitions and LASSO bi-plots from Climate Change Simulations; Supplemental Materials S1: Table S1. Definition and sources of global climate change model acronyms; Methods S1 in Supplemental Materials S1: Simulation of cattle grazing in SWAT; Table S1 in Supplemental Materials S1: WMOST data sources; Methods S2 in Supplemental Materials S2: Modifications to SWAT model for Upper Soldier Creek [40,76–82]. Methods S3: WMOST data sources and calibration [83–85]. Supplemental Materials S5. Riparian bank stabilization costs and efficiencies [23,32,41,55,86–88]. Supplemental Materials S6: Stables 6.1–6.2 Summary of WMOST Runs Supplemental Materials S7: Files (ASCII) S1: Future climate time series; Supplemental Material S8 (spreadsheet). Calculation of inputs for optimization of sizing of off-channel wetland (WMOST reservoir); Supplemental Materials S9: ScenCompare files for TP climate change scenarios.

## Figures and Tables

**Table 1.** Target loads for WMOST optimization runs.

Parameter	Units	Daily/Annual	Target
TSS	mT	Annual	8618
TSS	mg/L	Daily average	100 at flows <28.3 m^3^ s^*−*1^
TP	kg	Annual	40,585

**Table 2.** SWAT Model Calibration for Soldier Creek, Subbasins 1–7. Variable_n refers to calibration results for subbasin n, from 1 (headwaters of Upper Soldier Creek) to 3 (mouth of Upper Soldier Creek) to 7 (Soldier Creek outlet). NS = Nash–Sutcliffe coefficient, MSE = mean squared error, PBIAS = % bias, KGE = Kling–Gupta Efficiency, sim = simulated value, obs = observed value, StdDev = standard deviation.

Variable	Units	r^2^	NS	MSE	PBIAS	KGE	Mean_sim (Mean_obs)	StdDev_sim (StdDev_obs)
Discharge_1	mm	0.53	0.52	1.00 *×* 10^1^	*−*2.4	0.54	0.74 (0.73)	2.93 (4.61)
Discharge_3	mm	0.47	0.47	1.10 *×* 10^2^	*−*20.9	0.49	3.01 (2.49)	9.37 (14.20)
Discharge_7	mm	0.63	0.63	2.00 *×* 10^2^	*−*36.1	0.53	6.35 (4.67)	17.83 (23.05)
TSS load _3	mT/km^2^/day	0.1	0.06	9.60 *×* 10^2^	56.5	*−*0.1	5.39 (1123.09)	10.89 (2890.29)
Suspended sediment load_3	mT/km^2^/day	0.2	0.11	9.00 *×* 10^2^	*−*70.4	*−*0.11	1913.25 (1123.09)	1019.68 (2890.29)
TSS_7	mg/L	0.26	*−*0.01	1.10 *×* 10^7^	88	*−*0.41	6314.91 (581.88)	4242.00 (3329.33)
Total P_3	kg P/day	0.76	*−*0.48	1.40 *×* 10^3^	*−*58.1	*−*0.13	17.04 (10.78)	60.21 (30.64)
Total N_3	kg P/day	0.5	0.39	2.00 *×* 10^4^	18.2	0.66	69.96 (85.53)	187.38 (182.12)
Total N_7	kg N/day	0.7	0.68	5.40 *×* 10^5^	28	0.59	353.27 (490.80)	967.76 (1299.00)

**Table 3.** Summary of WMOST optimization and simulation results for Upper Soldier Creek.

Pollutant	Target Component	Year	Annual Load Target Achieved?	Daily Load Target Achieved?	BMPs/ACPs to Implement	Costs (million $)			Comments
						Infrastructure	BMP/ACP	Total	
TP	Upland loads	2012	Yes	Yes		$1.107		$1.107	

TP	Upland loads	2014	Yes	Yes	3.1 km^2^ developed area treated grassed swales with underdrains		$1.059		
TP	Upland loads				71.3 km^2^ cropland treated with contouring		$0.108		
TP	Upland loads					$2.058		$3.224	

TP	Upland loads	2015	Yes	Yes					
TP	Upland loads				8.9 km^2^ developed area treated with sand filter with underdrains		$1.772		
TP	Upland loads				71.3 km^2^ cropland treated with contouring		$0.108		
TP	Upland loads					$1.130		$3.01	

TP	Upland loads	2015	Yes	Yes	9.9 km^2^ developed area treated with wet ponds		$1.95		Groundwater supplement required
TP	Upland loads				71.3 km^2^ cropland treated with contouring		$0.108		
TP	Upland loads					$1.130		$3.19	

TSS	Upland loads	2012	Yes	Yes	35.2 km^2^ cropland treated with contouring		$0.052		
					0.02 km^2^ treated with agricultural constructed wetland		$0.00046		
						$1.107		$1.156	

TSS	Upland loads	2014	5.8 *×* target	No	13.3 km^2^ developed area treated with grassed swales with drains		$4.621		
					1.1 km^2^ cropland treated with terracing + no till 3328 m of bank		$0.009		
					stabilization/riparian restoration		$10.43		
					Cattle exclusion from 95.1 km^2^		$0.94		
								$17.000	Cheapest solution with bank stabilization

TSS	Upland + bank erosion	2014	10 *×* target	No	Off-channel wetland; 3421,255 m^3^ storage		$34		Additions to handle excess flows

## Data Availability

The calibrated SWAT model for Upper Soldier Creek will be archived in CUAHSI’s HydroShare, available at: http://www.hydroshare.org/resource/22e72bca6f754ac0a2ccc831ced21f22, accessed on 25 July 2025. WMOST optimization and simulation files are available in the Supplementary Materials. Underlying optimization software programs are available at www.epa.gov/ceam/wmost. Data underlying figures in this manuscript are available at: Figure 4: https://doi.org/10.23719/1532302 (accessed on 25 July 2025), Figure 5: https://doi.org/10.23719/1532301 (accessed on 25 July 2025), Table 2: https://doi.org/10.23719/1532303 (accessed on 25 July 2025).

## Abbreviations

ACP: Agricultural Conservation Practices
BASINS: Better Assessment Science Integrating Point and Nonpoint Sources
BMP: Best management practice
CAM-WRAP: Climate Assessment Module Wrapper
CAT: Climate Assessment Tool
CMhyd: Climate Model Data for Hydrologic Modeling
CMIP5: Coupled Model Intercomparison Project Phase 5
CN: Curve number
CUAHSI: Consortium of Universities for the Advancement of Hydrologic Science, Inc.
ECHO: Enforcement and Compliance History Online
HAWQS: Hydrologic and Water Quality System
HCAM: Hydrologic Comparison Assessment Module
HRU: Hydrologic Response Unit
HSPF: Hydrological Simulation Program-FORTRAN
HUC: Hydrologic Unit Code
ICF: Consulting company
KDHE: Kansas Department of Health and the Environment
KGE: Kling-Gupta Efficiency
LASSO: Locating and Selecting Scenarios Online
LOCA: Localized Constructed Analogs
LSRB: Le Sueur River Basin
MDE: Maryland Department of the Environment
MDPI: Multidisciplinary Digital Publishing Institute
MOEA: Multi-objective evolutionary algorithm
MOS: Margin of safety
N: Nitrogen
NEOS: Network-Enabled Optimization System
NLCD: National Landcover Dataset
NSE: Nash-Sutcliffe Efficiency
O&M: Operation and maintenance
ORISE: Oak Ridge Institute for Science and Education
P: Phosphorus
PRISM: Parameter-elevation Relationships on Independent Slopes Model
RCP: Representative Concentration Pathway
RMSE: Root mean square error
RSR: RMSE-observations standard deviation ratio
SPARROW: SPAtially-Referenced Regression On Watershed attributes
SWAT: Soil Water Assessment Tool
SWMM: Stormwater Management Model
SWMM-CAT: Stormwater Management Model Climate Assessment Tool
TMDL: Total Maximum Daily Load
TN: Total nitrogen
TP: Total phosphorus
TSS: Total suspended solids
USA: United States of America
US EPA: United States Environmental Protection Agency
USC: Upper Soldier Creek
USGS: United States Geological Survey
WMOST: Watershed Management Optimization Support Tool
WRAPS: Watershed Restoration Protection Strategy
WQ: Water quality
WWTP: Wastewater treatment plant
