# Supplementary material for: Optimization of Agricultural and Urban BMPs to Meet Phosphorus and Sediment Loading Targets in the Upper Soldier Creek, Kansas, USA
Source: Water (Basel). Author manuscript; Available in PMC 2025 Sep 12. (PMC12425134; doi:10.3390/w17152265)
Supplement: Supplement1 — The following supporting information can be downloaded at: https://www.mdpi.com/article/10.3390/w17152265/s1, Figure S1a–e in Supplemental Materials S1: Climate change scenario definitions and LASSO bi-plots from Climate Change Simulations; Supplemental Materials S1: Table S1. Definition and sources of global climate change model acronyms; Methods S1 in Supplemental Materials S1: Simulation of cattle grazing in SWAT; Table S1 in Supplemental Materials S1: WMOST data sources; Methods S2 in Supplemental Materials S2: Modifications to SWAT model for Upper Soldier Creek [40,76–82]. Methods S3: WMOST data sources and calibration [83–85]. Supplemental Materials S5. Riparian bank stabilization costs and efficiencies [23,32,41,55,86–88]. Supplemental Materials S6: Stables 6.1–6.2 Summary of WMOST Runs Supplemental Materials S7: Files (ASCII) S1: Future climate time series; Supplemental Material S8 (spreadsheet). Calculation of inputs for optimization of sizing of off-channel wetland (WMOST reservoir); Supplemental Materials S9: ScenCompare files for TP climate change scenarios. [file NIHMS2101745-supplement-Supplement1.zip › Supplemental Materials S1/Supplemental Table S1.pdf]

Supplemental Table S1. Definition and sources of global climate change model acronyms

Modeling Center	Model	Institution
BCC	BCC-CSM1.1<br>BCC-CSM1.1(m)	Beijing Climate Center, China Meteorological Administration
CCCma	CanESM2	Canadian Centre for Climate Modelling and Analysis
CMCC	CMCC-CM<br>CMCC-CMS	Centro Euro-Mediterraneo per I Cambiamenti Climatici
CNRM-CERFACS	CNRM-CM5	Centre National de Recherches Meteorologiques / Centre Europeen de Recherche et Formation Avancees en Calcul Scientifique
CSIRO-BOM	ACCESS1.0<br>ACCESS1.3	CSIRO (Commonwealth Scientific and Industrial Research Organisation, Australia), and BOM (Bureau of Meteorology, Australia)
CSIRO-QCCCE	CSIRO-Mk3.6.0	Commonwealth Scientific and Industrial Research Organisation in collaboration with the Queensland Climate Change Centre of Excellence
EC-EARTH	EC-EARTH	EC-EARTH consortium
INM	INM-CM4	Institute for Numerical Mathematics
IPSL	IPSL-CM5A-LR<br>IPSL-CM5A-MR	Institut Pierre-Simon Laplace
LASG-CESS	FGOALS-g2	LASG, Institute of Atmospheric Physics, Chinese Academy of Sciences; and CESS, Tsinghua University
MIROC	MIROC5	Atmosphere and Ocean Research Institute (The University of Tokyo), National Institute for Environmental Studies, and Japan Agency for Marine-Earth Science and Technology
MIROC	MIROC-ESM<br>MIROC-ESM-CHEM	Japan Agency for Marine-Earth Science and Technology, Atmosphere and Ocean Research Institute (The University of Tokyo), and National Institute for Environmental Studies
MOHC (additional realizations by INPE)	HadGEM2-CC<br>HadGEM2-ES	Met Office Hadley Centre (additional HadGEM2-ES realizations contributed by Instituto Nacional de Pesquisas Espaciais)
MPI-M	MPI-ESM-LR<br>MPI-ESM-MR	Max Planck Institute for Meteorology (MPI-M)
MRI	MRI-CGCM3	Meteorological Research Institute
NASA GISS	GISS-E2-H<br>GISS-E2-R	NASA Goddard Institute for Space Studies
NCAR	CCSM4	National Center for Atmospheric Research
NCC	NorESM1-M	Norwegian Climate Centre
NIMR/KMA	HadGEM2-AO	National Institute of Meteorological Research/Korea Meteorological Administration

Modeling Center	Model	Institution
NOAA GFDL	GFDL-CM3	Geophysical Fluid Dynamics Laboratory
	GFDL-ESM2G	
	GFDL-ESM2M	
NSF-DOE-NCAR	CESM1(BGC)	National Science Foundation, Department of Energy, National Center for Atmospheric Research
	CESM1(CAM5)	
