# Supplementary material for: Optimization of Agricultural and Urban BMPs to Meet Phosphorus and Sediment Loading Targets in the Upper Soldier Creek, Kansas, USA
Source: Water (Basel). Author manuscript; Available in PMC 2025 Sep 12. (PMC12425134; doi:10.3390/w17152265)
Supplement: Supplement1 — The following supporting information can be downloaded at: https://www.mdpi.com/article/10.3390/w17152265/s1, Figure S1a–e in Supplemental Materials S1: Climate change scenario definitions and LASSO bi-plots from Climate Change Simulations; Supplemental Materials S1: Table S1. Definition and sources of global climate change model acronyms; Methods S1 in Supplemental Materials S1: Simulation of cattle grazing in SWAT; Table S1 in Supplemental Materials S1: WMOST data sources; Methods S2 in Supplemental Materials S2: Modifications to SWAT model for Upper Soldier Creek [40,76–82]. Methods S3: WMOST data sources and calibration [83–85]. Supplemental Materials S5. Riparian bank stabilization costs and efficiencies [23,32,41,55,86–88]. Supplemental Materials S6: Stables 6.1–6.2 Summary of WMOST Runs Supplemental Materials S7: Files (ASCII) S1: Future climate time series; Supplemental Material S8 (spreadsheet). Calculation of inputs for optimization of sizing of off-channel wetland (WMOST reservoir); Supplemental Materials S9: ScenCompare files for TP climate change scenarios. [file NIHMS2101745-supplement-Supplement1.zip › Supplemental Materials S2/Supplemental Materials S2.pdf]

# Supplemental Material 2: Modifications to SWAT model for Upper Soldier Creek

## Point source discharges

We assumed 57% of TP effluent from wastewater is soluble reactive phosphorus (P), 2.8% is organic P and 40% is particulate mineral P (total of 97% mineral P and 3% organic P) [1]. For the Potawatomie treatment plant, modeled discharge values were used in place of measured values because violations had been reported due to inadequate flow calibrations at the facility. We also added initial groundwater concentrations of 0.1 mg N/L for $NO_3$-N and 0.0433 mg P/L for soluble P to replace default SWAT values of zero.

## Baseline SWAT model set-up and calibration

The baseline SWAT model from HAWQS was modified to represent existing agricultural management activities. Information on existing agricultural conservation practices in USC (cover crops, residue and tillage management, contouring, terraces, filter strips, controlled stream access for cattle, managed grazing), as of 2015 were compiled from KDHE (Andrew S. Lyon, KDHE, pers. comm.). To model the effects of cattle grazing and associated BMPs, we used methodology described in Sheshukov et al. [2]; See Supplemental Material 2)). Following guidance in Waidler et al. [3], cover crops were implemented using the land use update (.lup) table in SWAT and modifying HRU fraction (HRU_FRC) to simultaneously convert area from converted crop land (CORN or SOY or CORNSOY combinations) to SOYWW (soy/winter wheat) or WWSY (winter wheat/soy) sequences.

The baseline SWAT model for USC, including existing BMPs (Contouring (3 $km^2$ or 736 acres treated), grassed waterways (12 $km^2$ or 2,965 acres), no till (4 $km^2$ or 966 acres), terracing (4 $km^2$ or 966 acres), vegetative filter strips (3 $km^2$ or 848 acres), created agricultural wetlands (0.1 $km^2$ or 36 acres treated) and cattle grazing), was calibrated using the Sequential Uncertainty Fitting version 2 (SUFI-2) algorithm [4]. SUFI-2 operates by performing several iterations in the calibration process, usually at least 100 iterations per variable being calibrated. We conducted calibrations in a piecewise manner, using standard calibration procedures for large watersheds [5],[6]. The model was first calibrated at a daily time step for flow using data from USGS gauging stations USGS 06889170, USGS 06889180, and USGS 06889200 for the period 1995 – 2015 until reasonable calibration statistics (Nash-Sutcliffe Efficiency (NSE) values close to 0.6) were achieved. Calibrations were conducted starting with upstream gages and proceeding downstream. In cases where the gauging station did not correspond to the outlet of a subbasin, the drainage area of the HUC12 was compared to the drainage area upstream of the observation point and a correction factor was applied. Calibration was then performed for sediment loads, and then finally for nutrient loads. TSS, TP, and total nitrogen (TN) data were downloaded from the USGS/US EPA Water Quality Portal [7], with TSS derived from the USGS, and TP and TN data collected by the USGS, KDHE, and Prairie Band of the Potawatomie. When insufficient matching measured flow values were available to convert measured TSS and TP concentrations to loads, we substituted modeled flow values for load calculations.

## Simulation of cattle grazing in SWAT

Hydrologic response units (HRUs) with land uses of WWHT, SGHY, RNGE, and HAY were assumed to have cattle grazing on them and assigned an average stocking rate of 43 animal units (AU)/mi$^2$ (stocking rate for nearby Upper Rock Creek from Middle Kansas WRAPS document), with grazing operations taking place from April 20 - October 15. The stocking rate was used to estimate manure deposition for pasture/hay as 0.743 kg dry weight of manure deposited daily per hectare, with associated nutrient fractions for NO3-N, organic N, soluble P, and organic P derived from Shesukov et al. (2016): 0.01 for NO3-N, 0.03 for organic N, 0.004 for soluble P, and 0.007 for organic P. Deposition was calculated using AU and the relationship between stocking rate and manure deposition (measured in oven-dry-weight of manure deposited in tons/AU/year) according to Gollehon et al. (2001). Values for biomass consumption, waste, and trampling per AU were adapted from Montana State University Forage Consumption guidance documents (http://animalrange.montana.edu/documents/extension/forageconsump.pdf). Pre-BMP grazing parameters on upland pasture were grazing days (GRZ_DAYS) = 178, manure identification number (MANURE_ID) = 44, dry weight biomass consumed (BIO_EAT; kg/ha/day) = 1.506142 kg/ha/day, biomass weight trampled daily (BIO_TRMP) = 0.376535 kg/ha/day, and dry weight manure deposited daily (MANURE_KG) = 0.74275573 kg/ha/day. In the absence of cattle management BMPs, cattle time allocation budgets were assumed to be: 14 minutes in the stream subarea, 67 minutes in the riparian subarea, and 1,359 minutes in the pasture subarea (Sheshukov et al. 2016). Manure additions directly to the stream were calculated separately and added to the lumped rangeland HRU in WMOST.

Following examples of cattle management BMPs in Sheshukov et al. (2016), scenarios with cattle management BMPs and land use changes were implemented to reflect changes in pasture usage and grazing operations to restrict cattle grazing. Grazing in or near stream and riparian zones is likely to increase loads. These “synthetic pasture scenarios” were built and calibrated at the watershed scale.

The Sheshukov methodology to model fencing and off-stream watering sites involves creating synthetic pastures constructed of three zones: an in-stream area (defined as S), a riparian zone (designated as R) and grazing area designated as G. Only one grazing zone was utilized in the application to the Upper Soldier Creek watershed, as there was very little variability in slope class in the watershed. The zonation of the grazing areas is meant to reflect both the installation of fencing which restricts access to stream bank and in-stream areas and forces utilization of off-stream watering sites, and installation of cattle exclusion fencing to restrict cattle access and grazing to only pasture areas. The implementation of these BMPs restricts the amount of in-stream time for cattle and associated direct deposition.

The distribution of existing forested and pasture HRUs and stream length that’s located in agricultural areas (adjacent to farming/cattle grazing operations) were used as the basis for designing the synthetic pasture. The total stream length in USC is approximately 406 kilometers, of which 152 run through agricultural/rangeland (KDHE WRAPS 9E). A 10-meter wide floodplain area and a 15-meter wide riparian buffer on each side of the stream were assumed to be present as in Sheshukov et al (2016). In-stream area accounted for 0.42% of the total study area, while riparian zones accounted for 1.26%.

In translating the Sheshukov approach to be applied in the USC watershed, we modified the watershed model in the following manner:

- The total number of hectares included under HRUs associated with cattle management were divided and split up into the three subareas listed above (S,R, and G). HRUS with land uses RNGE, WWHT, or SGHY were designated as pasture (HAY) and were designated as Grazing lands (G).
- Additional HRUs that did not meet the criteria listed above were assigned a stocking rate of 43 AU/hectare as described above.
- Cattle movement was simulated by removing cattle from the stream HRU and adjusting the stocking rates in the riparian and grazing HRUs based on the changes in percentage of time spent in each subarea.

Pollutant parameters at pasture edge were modified according to modifications made in Sheshukov et al. (2016). Buffer strips were implemented in the pasture design and BMPs implemented in the Cattle Scenario in Upper Soldier Creek. The pollutant parameters in the .chm and .mgt files were modified according to Table 1.

*Table 1: Calibration Values for Pollutant Parameters at Pasture Edge*

| Parameter | Description | Calibration Range | Calibrated Value |
|---|---|---|---|
| SOL_ORGN | Initial Organic Nitrogen conc. in top soil layer (ppm) | 85-800 | 500 |
| SOL_ORGP | Initial Organic Phosphorus conc. in top soil layer (ppm) | 1.5-500 | 400 |
| SOL_SOLP | Initial Soluble Phosphorus conc. in top soil layer (ppm) | 2.5-100 | 7.5 |
| BIOMIX | Biological Mixing Efficiency | 0.0-1.0 | 0.8 |
| BIO_MIN | Minimum Plant Biomass for Grazing (kg/ha) | 0-650 | 500 |

References


1. Gu, A.Z.; Liu, L.; Neethling, J.B.; Stensel, H.D.; Murthy, S. Treatability and Fate of Various Phosphorus Fractions in Different Wastewater Treatment Processes. *Water Sci. Technol.* **2011**, *63*, 804–810, doi:10.2166/wst.2011.312.
2. Sheshukov, A.Y.; Douglas-Mankin, K.R.; Sinnathamby, S.; Daggupati, P. Pasture BMP Effectiveness Using an HRU-Based Subarea Approach in SWAT. *J. Environ. Manage.* **2016**, *166*, 276–284, doi:10.1016/j.jenvman.2015.10.023.
3. Waidler, D.; White, M.; Steglich, E.; Wang, S.; Williams, J.; Jones, C.A.; Srinivasan, R. *Conservation Practice Modeling Guide for SWAT and APEX*; 2009;
4. Abbaspour, K.C. *SWAT-CUP: SWAT Calibration and Uncertainty Programs—a User Manual*; Swiss Federal Institute of Aquatic Science and Technology: Dübendorf, Switzerland, 2011;
5. Arnold, J.G.; Moriasi, D.N.; Gassman, P.W.; Abbaspour, K.C.; White, M.J.; Srinivasan, R.; Santhi, C.; D. Harmel, R.; van Griensven, A.; W. Van Liew, M.; et al. SWAT: Model Use, Calibration, and Validation. *Trans. ASABE* **2012**, *55*, 1491–1508, doi:10.13031/2013.42256.

6. Abbaspour, K.; Vaghefi, S.; Srinivasan, R. A Guideline for Successful Calibration and Uncertainty Analysis for Soil and Water Assessment: A Review of Papers from the 2016 International SWAT Conference. *Water* **2017**, *10*, 6, doi:10.3390/w10010006.
7. Environmental Protection Agency; United States Geological Survey Water Quality Portal 2013.
