# Supplementary material for: Optimization of Agricultural and Urban BMPs to Meet Phosphorus and Sediment Loading Targets in the Upper Soldier Creek, Kansas, USA
Source: Water (Basel). Author manuscript; Available in PMC 2025 Sep 12. (PMC12425134; doi:10.3390/w17152265)
Supplement: Supplement1 — The following supporting information can be downloaded at: https://www.mdpi.com/article/10.3390/w17152265/s1, Figure S1a–e in Supplemental Materials S1: Climate change scenario definitions and LASSO bi-plots from Climate Change Simulations; Supplemental Materials S1: Table S1. Definition and sources of global climate change model acronyms; Methods S1 in Supplemental Materials S1: Simulation of cattle grazing in SWAT; Table S1 in Supplemental Materials S1: WMOST data sources; Methods S2 in Supplemental Materials S2: Modifications to SWAT model for Upper Soldier Creek [40,76–82]. Methods S3: WMOST data sources and calibration [83–85]. Supplemental Materials S5. Riparian bank stabilization costs and efficiencies [23,32,41,55,86–88]. Supplemental Materials S6: Stables 6.1–6.2 Summary of WMOST Runs Supplemental Materials S7: Files (ASCII) S1: Future climate time series; Supplemental Material S8 (spreadsheet). Calculation of inputs for optimization of sizing of off-channel wetland (WMOST reservoir); Supplemental Materials S9: ScenCompare files for TP climate change scenarios. [file NIHMS2101745-supplement-Supplement1.zip › Supplemental Materials S3/Supplemental Materials S3.pdf]

Supplemental Materials:
Optimization of agricultural and urban BMPs to meet phosphorus and sediment loading targets in the Upper Soldier Creek, Kansas

Supplemental Material 3: WMOST data sources and calibration

Point sources:

Point sources were originally derived from EPA's Discharge Monitoring Reports but subsequent investigations found that the reported loads were erroneous due to a malfunctioning current meter at two WWTP facilities. Point source loads from these facilities were subsequently estimated using flow values reported after corrections were implemented and annual discharge values had stabilized (post 2015). These point sources were entered as "Other Surface Water Discharges" on the Surface Water tab.

Water demand:

Water demand was calculated for public, private and agricultural uses based on USGS Kansas Public Water Supply reports.

Water sources:

Drinking water for the city of Soldier and the Potawatomie Reservation and Casino complex are supplied by the Rural Water District 3 which derived its water from groundwater wells in the Delaware River Basin (based on descriptions on web site) around 2013 and subsequently primarily from the Banner Creek Reservoir, also in the Delaware River Basin. Thus, these water sources should be accounted for as interbasin transfers to prevent WMOST from withdrawing the drinking water from within the Upper Soldier Creek watershed. The capacity for surface and groundwater pumping in the watershed was set to zero and ITB supplies currently have no limits.

Private water sources to populations not served by public water (1080.6 – 140 (Soldier) – 438 (Reservation) = 502.6 should be accounted for as "Other GW withdrawal" on the groundwater tab. Assume same per capita use as public water users and multiply public demand x 502.6/578 = 0.8696 to get other GW withdrawal time series. Multiply other groundwater withdrawals by (100 - avg percent consumptive use)/100 to get returns to septic systems (other GW discharge) in Upper Soldier Creek.

Septic System Users

The 2010 population within the Upper Soldier Creek watershed is 1080.6 with 140 in Soldier and 1238 in the reservation boundaries of which 438 is in Upper Soldier Creek watershed. The percent resident population discharging to septic systems from private water sources should be (1080.6 - 140 - 438.3)*100/1080.6% = 46.5% but we assumed all public water customers in Soldier and the reservation are served by the sewer system and WWTPs so discharge to septic from PWS is zero percent.

From https://www.usbr.gov/gp/nkao/water_needs/kickapoo1.pdf

Potawatomi Nation water needs (current and future)

Period	Population	Use rate (gpcd)[1]	Average daily demand (gpd[2])	Commercial/casino demand (gpd)[3]	Total demand (gpd)
Current	1,625	115	186,875	50,250	237,125
2020	2,250	115	258,750	94,750	353,500
2040	2,935	115	337,525	94,750	432,275

[1] Gallons per capita per day – includes institutional, commercial, and industrial water supply needs except the casino and other large commercial water users.
[2] Gallons per day.
[3] This column represents the needs of large commercial water users. For the current period, the demand shown is for the existing casino.

Soldier use rate for 2015 (Public Supply Water Use in Kansas from https://www.sciencebase.gov/catalog/item/58bd9576e4b01a6517dc0cfd) is 50 gallons/capita/day. Variation from 2011 to 2015 was: 51, na, 47, 49, 50, and 49 gcd.

Prairie Band use rate is 115 gpcd. Casino demand for 2011 is estimated as (50,250 + 94,750)/2 = 72,500 gpd.
Example PWS estimate is (140 * 50) + (1238 * 115) + 72500 = 221870 gpd = 221870/1000000 = 0.2219 mgd. Annual gpcd is substituted for 50.
Assume water use from private wells for remainder of population in watershed is similar to Rural Water District JA-03 = 91 gpcd. 91 * (1080.6 - 140 - 438.3) = 45709.3 gpd = 45709.3/1000000 = 0.0457 mgd. Annual gcpd for Jackson County RWD 03 is substituted for 91.

Table 1. Sources of WMOST input data

Parameter	Source	Calculations
Runoff time series	Recalibrated SWAT model output	SAS program used to calculated area-weighted average runoff from individual HRUs in model
Recharge time series	Recalibrated SWAT model output	SAS program used to calculated area-weighted average runoff from individual HRUs in model
Runoff loading time series (TP, TSS)	Recalibrated SWAT model output	SAS program used to calculated area-weighted average runoff from individual HRUs in model
Recharge loading time series (TSS)	Set to zero	
Recharge loading time series (TP)	Recalibrated SWAT model output	SAS program used to calculated area-weighted average runoff from individual HRUs in model
Point Sources (Other Surface Water Discharges)	EPA Dicharge Monitoring Reports	For Potawatomie WWTP, early discharge volumes replaced with later stabilized estimates recorded after malfunctioning pump was corrected
Water demand (public, private, agricultural)	Lanning-Rush 2016, Lanning-Rush and Restrepo-Osorio 2017	

Parameter	Source	Calculations
Drinking water source (Interbasin transfers)		Drinking water for the city of Soldier and the Potawatomie Reservation and Casino complex are supplied by the Rural Water District 3 which derived its water from groundwater wells in the Delaware River Basin (based on descriptions on web site) around 2013 and subsequently primarily from the Banner Creek Reservoir, also in the Delaware River Basin. The capacity for surface and groundwater pumping in the watershed was set to zero. ITB supplies currently have no limits.
Private water sources (Other groundwater withdrawal)		Private water sources to populations not served by public water (502.6) accounted for as "Other GW withdrawal" on the groundwater tab. Assumed same per capita use as public water users and multiplied public demand x 502.6/578 = 0.8696 to get other GW withdrawal time series.
Septic system use	https://www.usbr.gov/gp/nkao/water_needs/kickapoo1.pdf	The 2010 population within the Upper Soldier Creek watershed is 1080.6 with 140 in Soldier and 1238 in the reservation boundaries of which 438 is in Upper Soldier Creek watershed. The percent resident population discharging to septic systems from private water sources should be (1080.6 - 140 - 438.3)*100/1080.6% = 46.5% but we assumed all public water customers in Soldier and the reservation are served by the sewer system and WWTPs so discharge to septic from PWS is zero percent.

Lanning-Rush, J.L., and Restrepo-Osorio, D.L, 2017, Public-Supply Water Use in Kansas, 2015: U.S. Geological Survey data release, https://doi.org/10.5066/F7F769SC.

Lanning-Rush, J.L., 2016, Public-supply water use in Kansas, 2014:
U.S. Geological Survey data release, http://dx.doi.org/10.5066/F7N29V1D.

Consumptive use estimates (assume zero consumptive use for low use month):

Estimation of PWS consumptive use (from https://dnr.mo.gov/mowaterplan/documents/2018-02-06-consumptiveslides.pdf)				
Month	% annual demand	Consumptive Use	Nonconsumptive Use	Fraction consumptive use
Jan	8	1	7	0.125
Feb	7	0	7	0.000
Mar	8	1	7	0.125
Apr	8	1	7	0.125
May	8	1	7	0.125
Jun	9	2	7	0.222
Jul	10	3	7	0.300
Aug	11	4	7	0.364
Sep	10	3	7	0.300
Oct	9	2	7	0.222

Nov	8	1	7	0.125
Dec	7	0	7	0.000

Stormwater BMP selection

There is no stormwater design manual for the state of Kansas so we consulted the design manual for nearby Topeka, KS [1]. The design manual only allows stormwater BMPs without underdrains on well-drained soils (e.g., Hydrologic Soil Group A or B) so we only evaluated biofiltration with underdrain and wet ponds (grassed swale with underdrains and extended dry detention basin) as the two urban BMP options as potential green and gray infrastructure practices. Topeka has a designated water quality volume for treatment based on a 1.37 inch storm so this was used as the design depth.

Cattle grazing BMPs

The cattle restriction BMP for rangeland was implemented as a "direct reduction BMP" in WMOST under the water quality (WQ) BMPs option, with a TP load removal rate of 9.93% at $40.22/acre. Costs were derived from estimates of costs for components associated with cattle exclusion (e.g., fencing and provision of alternative water sources) derived from NRCS Payment Schedules for the state of Kansas (https://www.nrcs.usda.gov/state-offices/kansas/payment-schedule).

WMOST calibration

Both Type I and Type II calibrations were performed. Type I calibrations provide a check on overall water and pollutant balances and are carried out without any targets for flows or pollutant loads or management decision variables, with water demand explicitly modeled as a withdrawal time series, and wastewater discharge explicitly modeled as a point source discharge. Type II calibrations provide a check on the behavior of water supply/demand and are carried out without any targets for flows or pollutant loads or management decision variables. Type, timing, and magnitude of water withdrawals for drinking water are implicit decision variables. Water demand is entered as a time series separately for consumptive and nonconsumptive uses. Costs are entered to allow WMOST to allocate drinking water withdrawals between surface water, groundwater, and interbasin transfers to meet consumptive + nonconsumptive consumer demand, and wastewater discharge is estimated by WMOST based on consumptive use.

77. Lanning-Rush, J.L., and Restrepo-Osorio, D.L, 2017, Public-Supply Water Use in Kansas, 2015: U.S. Geological Survey data release, https://doi.org/10.5066/F7F769SC.

78. Lanning-Rush, J.L., 2016, Public-supply water use in Kansas, 2014:
U.S. Geological Survey data release, http://dx.doi.org/10.5066/F7N29V1D.

1. City of Topeka, Kansas 2023; Stormwater Development & Management. Stormwater Development & Management | Utilities, accessed 7/22/25.
