# Supplementary material for: Optimization of Agricultural and Urban BMPs to Meet Phosphorus and Sediment Loading Targets in the Upper Soldier Creek, Kansas, USA
Source: Water (Basel). Author manuscript; Available in PMC 2025 Sep 12. (PMC12425134; doi:10.3390/w17152265)
Supplement: Supplement1 — The following supporting information can be downloaded at: https://www.mdpi.com/article/10.3390/w17152265/s1, Figure S1a–e in Supplemental Materials S1: Climate change scenario definitions and LASSO bi-plots from Climate Change Simulations; Supplemental Materials S1: Table S1. Definition and sources of global climate change model acronyms; Methods S1 in Supplemental Materials S1: Simulation of cattle grazing in SWAT; Table S1 in Supplemental Materials S1: WMOST data sources; Methods S2 in Supplemental Materials S2: Modifications to SWAT model for Upper Soldier Creek [40,76–82]. Methods S3: WMOST data sources and calibration [83–85]. Supplemental Materials S5. Riparian bank stabilization costs and efficiencies [23,32,41,55,86–88]. Supplemental Materials S6: Stables 6.1–6.2 Summary of WMOST Runs Supplemental Materials S7: Files (ASCII) S1: Future climate time series; Supplemental Material S8 (spreadsheet). Calculation of inputs for optimization of sizing of off-channel wetland (WMOST reservoir); Supplemental Materials S9: ScenCompare files for TP climate change scenarios. [file NIHMS2101745-supplement-Supplement1.zip › Supplemental Materials S4/Supplemental Materials S4.pdf]

Supplemental Materials: Optimization of agricultural and urban BMPs to meet phosphorus and sediment loading targets in the Upper Soldier Creek, Kansas

Supplemental Material 4: Modifications to .mgt and .ops files to simulate ACPs

The approach below for modifying .mgt and .ops files to represent agricultural BMP implementation was updated to reflect changes in parameters that occurred as the result of the SWAT-CUP calibration process.

Terraces:

Terraces were added to the SWAT model by editing the operations (.ops) files via SWAT Editor. Terrace BMPs within a field are designed to intercept runoff and prevent erosion. Terraces are constructed across a slope contour at regular intervals, depending upon the slope class of the HRU. Terracing in SWAT is simulated by adjusting both erosion and runoff parameters. This includes the USLE Practice Factor (set to SWAT variables found in Table 20-6 of the SWAT User Manual), CN2 (set to SWAT variables defined in Table 20-1 of the SWAT user manual), and the average subbasin slope lengths (sub_slp), which were adjusted to simulate the effects of terracing. All values used were default SWAT values for designated land use and gently sloping landscapes (0-2% slope). Terracing was implemented in HRUs that included cropland, including CORN, SOYC, and SOYB,SOYC in subbasins 1, 2, and 3.

	Hydrologic Soil Group				
Parameter	B	C	D	SWAT category	SWAT Model Land Use
CN	71	78	81	row crop	CORN
CN	67	76	80	legumes	SOYB, ALFA
CN	70	78	81	small grains	BERM,HAY,SGHY,WWHT
TERR_P	0.12	0.12	0.12	All	All
TERR_SL	7.5	7.5	7.5	All	All

Grassed waterways operational parameters

Parameter	Value
MGT_OP	7
GWATI	1
GWATN	0.35
GWATSPCON	0.005
GWATD	0.46875
GWATW	10

Contouring operational parameters

			Hydrologic Soil Group		
	Category	Crop	B	C	D
MGT_OP			3	3	3
CONT_CN	row crop	CORN	75	82	86
CONT_CN	legumes	SOYB,ALFA	69	78	83
CONT_CN	small grains	BERM,HAY,SGHY,WWHT	73	81	84
CONT_P			0.9	0.9	0.9

No Till

SWAT parameter	Value
MGT_OP (*.mgt)	6
TILL_ID.mgt	ZEROTILL
CNOP.mgt	reduced by 3
ITNUM (till.dat)	4
EFFMIX (till.dat)	0.05
DEPTIL (till.dat)	25

Vegetative Filter Strip Operational Parameters

Parameter	Value
MGT_OP	4
VFSCON.ops	0.5
VFSRATIO.ops	50
VFSCH.ops	0

No Till practices are implemented in SWAT by updating the tillage records in the operation schedule (… = 6) (part 2 of .mgt files) to reflect no till practices (TILLAGE_ID = 4). CNOP is calculated by subtracting 2 or 3 from the curve number for condition II (CN2) to allow SWAT to switch curve numbers when tilling starts.
