# Supplementary material for: Optimization of Agricultural and Urban BMPs to Meet Phosphorus and Sediment Loading Targets in the Upper Soldier Creek, Kansas, USA
Source: Water (Basel). Author manuscript; Available in PMC 2025 Sep 12. (PMC12425134; doi:10.3390/w17152265)
Supplement: Supplement1 — The following supporting information can be downloaded at: https://www.mdpi.com/article/10.3390/w17152265/s1, Figure S1a–e in Supplemental Materials S1: Climate change scenario definitions and LASSO bi-plots from Climate Change Simulations; Supplemental Materials S1: Table S1. Definition and sources of global climate change model acronyms; Methods S1 in Supplemental Materials S1: Simulation of cattle grazing in SWAT; Table S1 in Supplemental Materials S1: WMOST data sources; Methods S2 in Supplemental Materials S2: Modifications to SWAT model for Upper Soldier Creek [40,76–82]. Methods S3: WMOST data sources and calibration [83–85]. Supplemental Materials S5. Riparian bank stabilization costs and efficiencies [23,32,41,55,86–88]. Supplemental Materials S6: Stables 6.1–6.2 Summary of WMOST Runs Supplemental Materials S7: Files (ASCII) S1: Future climate time series; Supplemental Material S8 (spreadsheet). Calculation of inputs for optimization of sizing of off-channel wetland (WMOST reservoir); Supplemental Materials S9: ScenCompare files for TP climate change scenarios. [file NIHMS2101745-supplement-Supplement1.zip › Supplemental Materials S5/Supplemental Materials S5.pdf]

Supplemental Materials:
Optimization of agricultural and urban BMPs to meet phosphorus and sediment loading targets in the Upper Soldier Creek, Kansas

Supplemental Materials 5: Riparian bank stabilization costs and efficiencies

Summary

Based on the following calculations, bank erosion inputs could account for 90% of estimated SWAT TSS loads and 25% of estimated SWAT TP loads. Our best estimate of average annual bank erosion is 8982.91 tons TSS/yr based on:

a) the median bank recession rate for mid-summer 2007 thru mid-summer 2010 for nonforested banks (1.5 ft/yr) (Sass 2001)
b) the length of eroding banks needing restoration from the Middle Kansas WRAP (30,919 ft; Stakeholder Leadership Team 2011),
c) a bank height of 9 ft calculated based on the relationship between discharge and channel width at the Soldier Creek gaging station near Delia (station 06889200; Juracek 2002), and
d) a soil bulk density for grazed pasture (.76 Mg/m3; Murphy et al. 2009)

We applied an average streambank P content for a nearby watershed (423.3 g P/1000kg soil; Juracek and Ziegler 2009) can be applied to the streambank erosion estimates to come up with a streambank load for total P for those hotspot erosion segments: 3802.765 kg P/yr. Applying a low-end bank erosion rate for remaining restorable stream segments (0.5 ft/yr) yields additional loads of 1,084 tons TSS/yr and 458.9496 kg P/yr. Streambank erosion will vary from year to year based on peak flows and soil saturation so we compared these with SWAT total load estimates for the same time period to come up with a fraction of total load related to bank erosion, and assumed that fraction was constant over time.

The cost for restoring those segments is estimated at $992.72/ft ($71.50/ft for stabilization (Powell 2017) + $921.22/ft for riparian restoration of 30m buffer for long-term stability (Kansas EQIP payment schedule). There are additional stream segments totalling 11,195 ft that could be subject to a dual restoration of riparian buffers plus creation of two-stage ditches for $20/ft (which create mini-floodplains within incised channels;) at a total cost of $941.22. If we have to lump those two strategies (stabilization + riparian and two-stage ditches + riparian) into a single BMP within WMOST the weighted average cost would be $954.91/ft.

Expected TSS and TP removal efficiency for the streambank stabilization is 85% (Stakeholder Leadership Team 2011) and for the two stage ditch + riparian restoration is 79% (Witter et al. 2011). A weighted average for the combined BMP is 83%.

References

Juracek, K.E. 2002. Historical Channel Change Along Soldier Creek, Northeast Kansas. U.S. Geological Survey. Lawrence, KS. Water-Resources Investigations Report 02–4047.

Juracek, K.E. and A.C. Ziegler. 2009. Estimation of sediment sources using selected chemical tracers in the Perry lake basin, Kansas, USA. International Journal of Sediment Research 24: 108–125.

Murphy, C.A., B.L. Foster, M.E. Ramspott, and K. Price. 2004. Grassland management and soil bulk density. Transactions of the Kansas Academy of Science 107(Apr 2004):45-54. DOI 10.1660/0022-8443(2004)107[0045:GMEOSB]2.0.CO;2

Powell, A. 2017. Delaware River Watershed Streambank Erosion Assessment: ArcGIS® Comparison Study: 1991, 2002, 2003 vs. 2015 Aerial Photography. Updated Draft April 2017. Kansas Water Office, KS.

Sass, C. 2011. Evaluation and Development of Predictive Streambank Erosion Curves for Northeast Kansas Using Rosgen's "Bancs" Methodology. PhD thesis. Univ of Kansas.

Stakeholder Leadership Team. 2011. Watershed Restoration and Protection Strategy Middle Kansas Watershed July 18, 2011. Available at: https://kswraps.org/wp-content/uploads/2020/10/middlekansas_plansummary_0.pdf.

Witter,J., J. D'Ambrosio, J. Magner, A. Ward and B. Wilson. 2011. Economics of Implementing Two-stage Channels. Dept. of Food, Agricultural and Biological Engineering, The Ohio State University, Dept. of Bioproducts & Biosystems Engineering, University of Minnesota. https://wrl.mnpals.net/islandora/object/WRLrepository%3A3542
