# Supplementary material for: Optimization of Agricultural and Urban BMPs to Meet Phosphorus and Sediment Loading Targets in the Upper Soldier Creek, Kansas, USA
Source: Water (Basel). Author manuscript; Available in PMC 2025 Sep 12. (PMC12425134; doi:10.3390/w17152265)
Supplement: Supplement1 — The following supporting information can be downloaded at: https://www.mdpi.com/article/10.3390/w17152265/s1, Figure S1a–e in Supplemental Materials S1: Climate change scenario definitions and LASSO bi-plots from Climate Change Simulations; Supplemental Materials S1: Table S1. Definition and sources of global climate change model acronyms; Methods S1 in Supplemental Materials S1: Simulation of cattle grazing in SWAT; Table S1 in Supplemental Materials S1: WMOST data sources; Methods S2 in Supplemental Materials S2: Modifications to SWAT model for Upper Soldier Creek [40,76–82]. Methods S3: WMOST data sources and calibration [83–85]. Supplemental Materials S5. Riparian bank stabilization costs and efficiencies [23,32,41,55,86–88]. Supplemental Materials S6: Stables 6.1–6.2 Summary of WMOST Runs Supplemental Materials S7: Files (ASCII) S1: Future climate time series; Supplemental Material S8 (spreadsheet). Calculation of inputs for optimization of sizing of off-channel wetland (WMOST reservoir); Supplemental Materials S9: ScenCompare files for TP climate change scenarios. [file NIHMS2101745-supplement-Supplement1.zip › Supplemental Materials S7/Readme.pdf]

Future climate time series

File naming:

Precmm_fut*n*_*AA*14521.csv = Precipitation time series for Upper Soldier Creek Subbasin *n* for RCP 4.5 climate change scenario based on *AA* model and future years of 2021 – 2050

tempdegc_futAA.csv – Air temperature time series for Upper Soldier Creek based on AA climate change model

Precipitation File Variables

Precipmm – Current daily precipitation (mm)

Julian – Julian day

MO - Month

DA – Day of month

YR - Year

Season deltachg – Multiplier to convert current precipitation to future precipitation

Precipmm_fut – Future daily precipitation (mm)

Temperature File Variables

yrjulian	Year + Julian day
YR	Year
MONTH	Month
DA	Day of month
avtemp1	Average of subbasin 1 min and max daily temperature (degrees C, current)
avtemp2	Average of subbasin 2 min and max daily temperature (degrees C, current)
avtemp3	Average of subbasin 3 min and max daily temperature (degrees C, current)
AA18716t_degc	Historic modelled temperature (deg C) under AA model, 1987 - 2016
AA14521t_degc	Projected temperature (deg C) under AA RCP 4.5 model for 2021 - 2050
AA14551t_degc	Projected temperature (deg C) under AA RCP 4.5 model for 2051 - 2080
AA18521t_degc	Projected temperature (deg C) under AA RCP 8.5 model for 2021 - 2050
AA18551t_degc	Projected temperature (deg C) under AA RCP 8.5 model for 2051 - 2080
delta_AA14521t	Projected change in temperature (deg C) under AA model, RCP 4.5, 2021 - 2050
delta_AA14551t	Projected change in temperature (deg C) under AA model, RCP 4.5, 2051 - 2080
delta_AA18521t	Projected change in temperature (deg C) under AA model, RCP 8.5, 2021 - 2050
delta_AA18551t	Projected change in temperature (deg C) under AA model, RCP 8.5, 2051 - 2080
avtemp_AAnRRYYt	Projected average air temperature (deg C) under AA model for subbasin n, RCP RR (4.5 or 8.5), Years 21 (2021 - 2050) or 51 (2051 - 2080)
mintemp_AAnRRYYt	Projected minimum air temperature (deg C) under AA model for subbasin n, RCP RR (4.5 or 8.5), Years 21 (2021 - 2050) or 51 (2051 - 2080)
maxtemp_AAnRRYYt	Projected maximum air temperature (deg C) under AA model for subbasin n, RCP RR (4.5 or 8.5), Years 21 (2021 - 2050) or 51 (2051 - 2080)

See Supplemental Materials 1 for climate change model (AA) definitions
