# Supplementary material for: Optimization of Agricultural and Urban BMPs to Meet Phosphorus and Sediment Loading Targets in the Upper Soldier Creek, Kansas, USA
Source: Water (Basel). Author manuscript; Available in PMC 2025 Sep 12. (PMC12425134; doi:10.3390/w17152265)
Supplement: Supplement1 — The following supporting information can be downloaded at: https://www.mdpi.com/article/10.3390/w17152265/s1, Figure S1a–e in Supplemental Materials S1: Climate change scenario definitions and LASSO bi-plots from Climate Change Simulations; Supplemental Materials S1: Table S1. Definition and sources of global climate change model acronyms; Methods S1 in Supplemental Materials S1: Simulation of cattle grazing in SWAT; Table S1 in Supplemental Materials S1: WMOST data sources; Methods S2 in Supplemental Materials S2: Modifications to SWAT model for Upper Soldier Creek [40,76–82]. Methods S3: WMOST data sources and calibration [83–85]. Supplemental Materials S5. Riparian bank stabilization costs and efficiencies [23,32,41,55,86–88]. Supplemental Materials S6: Stables 6.1–6.2 Summary of WMOST Runs Supplemental Materials S7: Files (ASCII) S1: Future climate time series; Supplemental Material S8 (spreadsheet). Calculation of inputs for optimization of sizing of off-channel wetland (WMOST reservoir); Supplemental Materials S9: ScenCompare files for TP climate change scenarios. [file NIHMS2101745-supplement-Supplement1.zip › Supplemental Materials S9/File Structure Description.pdf]

Climate_45_StreamStab File Structure

1. CAM_WRAP
    a. Outputs
        i. Avg Reduction
            1. CAMWRAP outputs for the average reduction streambank stabilization BMP
        ii. Min Reduction
            1. CAMWRAP outputs for the minimum reduction streambank stabilization BMP
2. HCAM
    a. Inputs
        i. Time Series
            1. SWAT loading time series as inputs to HCAM
        ii. BMP-Config.csv
        iii. HRU-Config.csv
        iv. HRU-Reference.csv
    b. Outputs
        i. HCAM output files
3. HCAM-R
    a. Inputs
    b. Outputs
        i. Avg Reduction
            1. QA
                a. Individual scenario files covering LRe, LRu, QRu variables for QA purposes
            2. HCAM-R outputs for the average reduction streambank stabilization BMP
        ii. Min Reduction
            1. QA
                a. Individual scenario files covering LRe, LRu, QRu variables for QA purposes
            2. HCAM-R outputs for the minimum reduction streambank stabilization BMP
4. NEOS_Results
    a. Avg Reduction
        i. NEOS results from the average reduction streambank stabilization BMP model
    b. Min Reduction
        i. NEOS results from the minimum reduction streambank stabilization BMP model
5. R_Programs
    a. Avg reduction
        i. 01_HP_HCAM_v5.R
        ii. 02_HP_HCAM_v7.R
        iii. CAMWRAP_V4.R
    b. Min reduction
        i. 01_HP_HCAM_v5.R
        ii. 02_HP_HCAM_v7.R
        iii. CAMWRAP_V4.R

6. Results
    a. wmostscencompare_streamstab_avgred_4-14-23.xlsx
    b. wmostscencompare_streamstab_minred_4-14-23.xlsx
7. WMOST_Models
    a. Avg Reduction
        i. WMOST Model
        ii. Model and Data Shell FIles
    b. Min Reduction
        i. WMOST Model
        ii. Model and Data Shell FIles
    c. Results_Processing
        i. Avg Reduction
            1. SpecsResults files for average reduction scenarios
        ii. Min Reduction
            1. SpecsResults files for minimum reduction scenarios
