# Supplementary material for: Optimization of Agricultural and Urban BMPs to Meet Phosphorus and Sediment Loading Targets in the Upper Soldier Creek, Kansas, USA
Source: Water (Basel). Author manuscript; Available in PMC 2025 Sep 12. (PMC12425134; doi:10.3390/w17152265)
Supplement: Supplement1 — The following supporting information can be downloaded at: https://www.mdpi.com/article/10.3390/w17152265/s1, Figure S1a–e in Supplemental Materials S1: Climate change scenario definitions and LASSO bi-plots from Climate Change Simulations; Supplemental Materials S1: Table S1. Definition and sources of global climate change model acronyms; Methods S1 in Supplemental Materials S1: Simulation of cattle grazing in SWAT; Table S1 in Supplemental Materials S1: WMOST data sources; Methods S2 in Supplemental Materials S2: Modifications to SWAT model for Upper Soldier Creek [40,76–82]. Methods S3: WMOST data sources and calibration [83–85]. Supplemental Materials S5. Riparian bank stabilization costs and efficiencies [23,32,41,55,86–88]. Supplemental Materials S6: Stables 6.1–6.2 Summary of WMOST Runs Supplemental Materials S7: Files (ASCII) S1: Future climate time series; Supplemental Material S8 (spreadsheet). Calculation of inputs for optimization of sizing of off-channel wetland (WMOST reservoir); Supplemental Materials S9: ScenCompare files for TP climate change scenarios. [file NIHMS2101745-supplement-Supplement1.zip › Supplemental Materials S9/UpperSoldierCreek_T5_SummaryMemo_5-4-23.pdf]

Memorandum

To: Naomi Detenbeck and Marilyn ten Brink, U.S. Environmental Protection Agency, Office of Research and Development

From: Sam Ennett and Alyssa Le

Date: May 4, 2023

Re: WMOST Upper Soldier Creek Climate Batch Runs (Emission Scenario 4.5) (Task Order 72, Task 11)

1 Introduction

At EPA technical direction, ICF ran the Upper Soldier Creek case study through a suite of WMOST-related programs to test the robustness of potential management practices to meet total phosphorus loading targets under varying climate scenarios. To evaluate an appropriate starting point, ICF ran the case study specifications without the total phosphorus loading target for 14 different climate scenarios under the 4.5 emission scenario for the 2021-2050 future time frame through the SWAT Batch Climate Runs code, HCAM, HCAM-R, CAM-WRAP, NEOS, and ScenCompare. In other words, ICF ran the Upper Soldier Creek case study in simulation mode across a variety of climate conditions in order to screen for the effectiveness of management practice implementation at meeting the desired nutrient target. After evaluating the simulation results, ICF proposed testing a new set of management practices for model optimization under the 4.5 emission scenario for the 14 different climate scenarios. The following sections summarize the simulation and optimization run results.

2 Upper Soldier Creek Model Simulation Results

ICF was able to run the Upper Soldier Creek case study through all of the WMOST-related programming without any major issues. The majority of the scenario model simulations also optimized quickly on the NEOS server.[1] All of the solved model simulations were loaded into ScenCompare for evaluation.[2]

In order to identify relevant next steps to evaluate the robustness of management practices across varying climate scenarios, ICF analyzed "LSw1" variable values, which represent phosphorus loads in the surface water, across climate scenario model simulations. We compared the variable values against the daily and annual loading targets from a WMOST model provided by EPA, 1,039.6 lbs/day and 10,913 lbs/year respectively. All climate scenario model simulations resulted in at least one exceedance of the daily loading target and all climate scenarios exceeded the annual loading target (see Table 2-1). Daily and annual loading target exceedances are highlighted in red on the "Table_LSw1" tab of the ScenCompare

[1] The GFC scenario was unable to optimize on the NEOS server. ICF did attempt to debug this issue, but there were no obvious issues with model setup. It is possible that there is an initial model input value that is incongruous with the climate scenario input data, causing model failure. Given that the majority of the other climate scenario model simulations were able to solve, EPA and ICF agreed to proceed with simulation results evaluation and optimization.

[2] The ScenCompare file with all of the loaded results is included as supplementary material to this memo. Note: as part of the debugging process, ICF tried various initial groundwater concentration values. The majority of the climate scenario model simulations solved with an initial groundwater concentration of 0 mg/L. One model run with an initial groundwater concentration of 0.04 mg/L was included in the ScenCompare file for EPA's reference.

results comparison file for reference. GIH climate scenario model simulation results exceeded the daily loading target the most with 16 exceedances, while the FG climate scenario model simulation results exceeded the daily loading target the least with two exceedances. Exceedances above the daily loading target were on average about 400 pounds greater, although some model results had daily loadings that were around 1,300 pounds over the target. The annual target was exceeded by every climate scenario with the lowest total annual loadings of 29,643.58 pounds for climate scenario AC and the highest total annual loadings of 53,723.51 for climate scenario GIR. Overall, the annual exceedances ranged from triple to quintuple the target annual loading value. Given the difference between simulated loadings and the annual loading target, EPA and ICF agreed to move forward with additional testing to meet the daily loading target only.

Table 2-1: ScenCompare Climate Scenario Summary

Climate Scenario	Number of Daily Exceedances	Annual Total (lbs/year)
CE4521_0GW	8	36,909.13
CM4521_0GW	10	43,179.46
CMS4521_0GW	7	35,166.88
FG4521_0GW	2	32,142.59
GFE4521_0GW	12	42,916.68
GIH4521_0GW	16	48,507.7
GIR4521_0GW	15	53,723.51
HA4521_0GW	4	30,336.94
HA4521_OPT	0	27,692.19
IN4521_0GW	3	31,712.79
MI4521_0GW	13	50,508.02
MIC4521_0GW	11	52,759.65
MR4521_0GW	11	52,891.79
AC4521_0GW	4	29,643.58
CE4521_04GW	10	43,262.54
Baseline	0	10,913.00

Source: ScenCompare Analysis

3 Initial Optimization Results

ICF proceeded with the optimization of the four models with fewer than five daily exceedances (FG, HA, IN, and AC) using the daily loading target. Of these four models, only HA successfully optimized through NEOS. This is likely because the total annual load for the WMOST simulation using the HA climate scenario was the lowest of the four models.

The optimized cost to meet the daily loading target under the HA scenario is $2,158,447.45 compared to the baseline scenario cost of $3,224,165.49. The HA scenario model optimization cost is less than the baseline scenario cost because the baseline scenario included both daily and annual surface water loading

targets, requiring the implementation of the more expensive grass swale management practice.[3] Both scenarios had similar costs otherwise, including choosing a similar amount of land area to implement contouring practices. Examining the surface water loading between the two scenarios, the HA scenario's annual loading is 27,692.19 pounds, which is 16,779.19 pounds higher than the annual loading from the baseline scenario. The maximum daily loading rate in the optimized HA scenario is 1,039.6 pounds compared to the baseline scenario with a maximum daily loading rate of 900.73 pounds. The average daily loading rate for the optimized HA scenario is 75.87 pounds per day while the rate is 29.9 pounds per day for the baseline scenario.

4 Additional Optimization Results

As described in Section 3, the contouring, extended dry detention basin, and grass swale BMPS did not reduce loads enough to optimize even across the four climate scenarios with the fewest exceedances. ICF conducted a subsequent analysis into the sources of phosphorus in the modeled area to determine why the loading targets could not be met. We found that over 60 percent of the phosphorus loads entering the stream came from range and hay land uses. The following sections describe additional BMPs that were tested for their effectiveness at meeting the daily loading target.

4.1 Direct Reduction BMP

To investigate the amount of load reductions needed to produce a successful optimization, we added a direct reduction BMP to the optimization model that represents practices (*e.g.,* cattle removal from riparian areas or street sweeping in urban areas) that result in a percentage reduction of landscape loadings to the stream. We tested various reduction percentages, including 80 and 95 percent reductions on range only and 95 percent reduction on range and 60 percent reduction on hay. Even with a 95 percent reduction on range and a 60 percent reduction on hay, only four of the 14 climate scenarios optimized.

4.2 Streambank Stabilization BMP

After failing to achieve optimization with the direct reduction BMP, we implemented a streambank stabilization or streambank restoration BMP as bank erosion is a large contributor of phosphorus and sediment loads in this watershed. The WMOST streambank stabilization BMP requires three inputs: removal rate, cost per linear stream length, and maximum linear stream length. In order to take advantage of the climate scenario capabilities of HCAM-R and CAM-WRAP, ICF had to utilize a single set of parameter inputs for the streambank stabilization BMP. EPA sent the values for cost per linear stream length and maximum linear stream length as $954.91 per foot and 42,114 feet respectively. ICF calculated the removal rate of phosphorus using the equation below at the direction of EPA where 0.83 is the percent load reduction of the streambank stabilization BMP provided by EPA, and the 30,919 and 11,195 values refer to the length of eroding banks needing restoration from the Middle Kansas Watershed Restoration and Protection strategy (WRAP) and additional stream segments that could be subject to a dual restoration of riparian buffers, respectively.

$$Removal\ Rate\ of\ TP = \frac{Total\ baseline\ loads\ (varies\ by\ modeling\ time\ frame) * 0.83}{(30{,}919 +\ 11{,}195) * number\ of\ time\ steps\ in\ modeling\ time\ frame}$$

[3] The baseline scenario model that ICF received has internally inconsistent management practice names. In other words, the management practice names reflected on the Land Use page differ from those used on the Stormwater page. For the purposes of this memo, ICF used the names reflected on the Land Use page.

Since the removal rate depends on baseline loadings that differ across the climate scenarios, ICF tested two model scenarios: one using the average removal rate of TP across the 14 climate scenarios (299 lbs/ft/timestep) and one using the minimum removal rate of TP across the 14 climate scenarios (213 lbs/ft/timestep).

All 14 of the climate scenarios optimized with the daily loading target. A summary of the results of the modeled scenarios can be found in Table 5-1. Costs for the scenarios ranged from a minimum of $1,114,696.52 in the FG scenario to a maximum of $1,187,925.85 in the GIR scenario. This is far less than the baseline scenario cost of $3,224,165.49. The highly reduced cost is likely due to the large pound per time step load reduction of the streambank stabilization BMP. Only a relatively small length of stream stabilization was necessary to reach the loading targets which translates to a lower cost of implementation. All scenarios chose to implement the streambank stabilization BMP only and the costs are directly related to how many feet of bank stabilization were necessary.

Table 5-1: ScenCompare Streambank Stabilization Average and Minimum Reduction Summary[4]

Climate Scenario	Average Daily Loading (lbs/day)	Annual Total (lbs/year)	Length of Stabilization (ft)	Cost
AC4521	53.19	19,417.35	28.44	$1.14 Million
CE4521	63.74	23,268.07	41.32	$1.15 Million
CM4521	71.25	26,009.50	50.49	$1.16 Million
CMS4521	59.27	21,634.99	35.85	$1.15 Million
FG4521	54.31	19,824.25	0.05	$1.11 Million
GFC4521	72.35	26,410.33	51.83	$1.16 Million
GFE4521	71.108	25,954.69	50.3	$1.16 Million
GIH4521	83.9	30,623.68	65.92	$1.18 Million
GIR4521	92.76	33,858.89	76.74	$1.19 Million
HA4521	52.56	19,185.42	27.66	$1.14 Million
MR4521	55.12	20,121.37	30.79	$1.14 Million
MI4521	85.81	31,322.87	68.26	$1.18 Million
MIC4521	89.79	32,774.01	73.11	$1.18 Million
MR4521	89.19	32,555.11	72.38	$1.18 Million

Source: ScenCompare Analysis

[4] The difference between the annual total pounds for the average and minimum reduction scenarios for the streambank stabilization BMP are negligible (on the order of 10^{-6}) so results are reported together.
